# Phase III Trial: Single Low-Dose 5 mg Dexamethasone with NEPA for Preventing 168 h Nausea and Vomiting of Diverse Highly or Moderately Emetogenic Chemotherapy (LD-NEPA)

**DOI:** 10.3390/diseases14070231

**Published:** 2026-06-27

**Authors:** Yuting He, Xintian Huang, Kaiyi Rong, Yongxiang Hong, Fanzhuoran Lou, Bowen Zheng, Weijuan Tan, Quan Chen, Huibo Shi, Li Xiao

**Affiliations:** 1 Department of Oncology, Longyan First Affiliated Hospital of Fujian Medical University, Fujian Medical University, Longyan 364000, China; 24520221154629@stu.xmu.edu.cn; 2 Department of Oncology, Zhongshan Hospital of Xiamen University, School of Medicine, Xiamen University, Xiamen 361004, China; 24620192204532@stu.xmu.edu.cn (X.H.); 24520251154896@stu.xmu.edu.cn (K.R.); 24520231154759@stu.xmu.edu.cn (F.L.); 24520220157288@stu.xmu.edu.cn (B.Z.); tanwj102@163.com (W.T.); cq498348619@163.com (Q.C.); 3 The Graduate School, Fujian Medical University, Fuzhou 350122, China; 4 Institute of Organ Transplantation, Tongji Hospital, Tongji Medical College, Huazhong University of Science and Technology, Wuhan 430030, China

**Keywords:** chemotherapy-induced nausea and vomiting, corticosteroid-sparing strategy, antiemetic prophylaxis, complete response, non-inferiority trial

## Abstract

Background/Objectives: Reducing corticosteroid exposure has become an important objective in the management of chemotherapy-induced nausea and vomiting (CINV), given the dose-related toxicity of dexamethasone (DEX). This prospective phase III randomized (LD-NEPA: low-dose dexamethasone plus netupitant/palonosetron) study evaluated whether a reduced dose of 5 mg DEX could maintain antiemetic efficacy when combined with NEPA in patients receiving highly or moderately emetogenic chemotherapy (HEC/MEC). Methods: Adult patients were randomly assigned (1:1) to receive NEPA with either 5 mg DEX (5D group) or 8 mg DEX (8D group). Among 186 randomized patients, 180 were included in the analysis (89 in the 5D group and 91 in the 8D group). The primary endpoint was the complete response rate (CRR; no emesis and no rescue medication use) during the 0–168 h period. Secondary endpoints included phase-specific CRR, total control rate (TCR), complete control rate (CCR), daily incidence of nausea and vomiting, and safety outcomes. Results: The overall CRR was 78.7% in the 5D group and 70.3% in the 8D group. The between-group difference was 8.4% (95% CI: −1.5% to 19.0%), demonstrating the reduced-dose regimen was non-inferior, with the lower bound exceeding the prespecified −15% margin. Consistent results were observed across secondary endpoints. Treatment-related adverse events were mostly grade 1–2 and occurred with broadly comparable frequencies between the two groups. Conclusions: A single lower dose of 5 mg DEX is noninferior to an 8 mg dose combined with NEPA for preventing 168 h nausea and vomiting of diverse HEC/MEC, offering comparable efficacy while reducing corticosteroid exposure.

## 1. Introduction

Chemotherapy-induced nausea and vomiting (CINV) remains one of the most distressing adverse effects associated with cancer treatment, significantly impairing quality of life and treatment adherence [1,2]. Antineoplastic agents are classified according to emetogenic potential, with highly emetogenic chemotherapy (HEC) and moderately emetogenic chemotherapy (MEC) accounting for the majority of clinically significant CINV [3]. Current international antiemesis guidelines, including those from the American Society of Clinical Oncology (ASCO), National Comprehensive Cancer Network (NCCN), Multinational Association of Supportive Care in Cancer (MASCC), and European Society for Medical Oncology (ESMO), uniformly recommend a three-drug prophylactic regimen comprising a 5-HT3 receptor antagonist (5-HT3 RA), NK-1 receptor antagonist (NK-1 RA), and dexamethasone (DEX) for patients receiving MEC or HEC, with olanzapine augmentation considered for high-risk cases [4,5,6].

DEX plays a central role in antiemetic control, particularly in the delayed phase. However, its use is associated with a range of dose-dependent adverse effects, including hyperglycemia, sleep disturbance, mood changes, and immunologic alterations, which may occur even after short-term exposure [7,8]. These risks are of particular concern in patients with metabolic comorbidities, advanced age, or those receiving repeated chemotherapy cycles, where cumulative corticosteroid exposure becomes clinically relevant.

With the introduction of NK-1 RA–based regimens and fixed combinations such as netupitant–palonosetron (NEPA), there has been increasing interest in DEX-sparing strategies. Several randomized studies have demonstrated that reducing the duration of DEX administration, particularly beyond day 1, does not compromise antiemetic efficacy in selected settings [9,10,11,12]. However, the available evidence remains heterogeneous, especially regarding delayed-phase control and in high-risk populations. Consequently, current guidelines continue to recommend DEX as a backbone component of antiemetic regimens, particularly in HEC settings [4,5].

In this context, a stepwise dose-reduction approach may represent a more clinically prudent strategy than immediate omission. Identifying the lowest effective dose of DEX that maintains antiemetic efficacy while minimizing corticosteroid exposure is therefore an important and clinically relevant question.

NEPA, a fixed-dose combination of netupitant and palonosetron, provides sustained blockade of both NK-1 and 5-HT3 pathways and has demonstrated consistent efficacy across chemotherapy cycles [13,14,15,16]. Emerging evidence supports the feasibility of single-day DEX administration when combined with NEPA, although the optimal dose remains undefined [11,15,17,18].

Our previous retrospective study demonstrated that a single 8 mg dose of DEX combined with NEPA provided effective 168 h CINV control in patients receiving MEC/HEC with high-risk factors [19]. Building on these findings, the present phase III randomized trial (LD-NEPA: low-dose dexamethasone plus NEPA) was designed to evaluate whether a further reduction to a single 5 mg dose could maintain antiemetic efficacy while potentially reducing corticosteroid exposure.

The selection of 5 mg represents a pragmatic dose-de-escalation step rather than a biologically defined threshold. Because the antiemetic contribution of DEX within NEPA-based regimens has not been fully quantified, we considered complete omission or more aggressive dose reduction insufficiently supported by available evidence at the time of study design. Accordingly, a 5 mg dose was chosen to retain a meaningful day-1 corticosteroid component while achieving a substantial reduction in overall DEX exposure compared with the institutional 8 mg regimen.

## 2. Methods

### 2.1. Study Design

This was a single-center, phase III, open-label, randomized, controlled non-inferiority trial conducted at Zhongshan Hospital of Xiamen University between 1 October 2024 and 1 August 2025. The trial was conducted in accordance with the Declaration of Helsinki and Good Clinical Practice guidelines and was approved by the institutional ethics committee (approval No. XMZSYY-AP-SC-12-03; approval date: 1 August 2024). All participants provided written informed consent before enrollment. The study was registered at the Chinese Clinical Trial Registry on 5 September 2024 (ChiCTR2400089311) [20]. This study was reported in accordance with the CONSORT 2025 guidelines for randomized trials. A completed CONSORT checklist, along with the full trial protocol and SPIRIT checklist, has been provided as Supplementary Materials.

At the time of study design, a single-dose 8 mg DEX regimen combined with NEPA had already been adopted in our institution as a pragmatic antiemetic strategy based on prior clinical experience and retrospective data. Therefore, the present trial was designed to evaluate further dose reduction (5 mg) relative to this locally established regimen.

### 2.2. Participants

Eligible patients were adults aged 18–75 years with histologically confirmed solid malignancies who were scheduled to receive MEC or HEC, as defined by standard guideline classifications.

Key inclusion criteria included: absolute neutrophil count of at least 1.5 × 10^9^/L, platelet count minimum 100 × 10^9^/L, Alanine Aminotransferase (ALT)/Aspartate Aminotransferase (AST) levels not exceeding three times the upper limit of normal (five times for hepatic metastases), creatinine clearance of 60 mL/min or higher, Eastern Cooperative Oncology Group (ECOG) performance status of 0–1, and minimum 12-week life expectancy. All participants provided written informed consent and were screened by board-certified medical oncologists with substantial clinical experience.

Risk stratification was performed at two levels. First, patients were classified according to the emetogenic potential of the planned chemotherapy regimen as receiving either HEC or MEC. Patients receiving HEC were considered high risk based on the chemotherapy regimen. Second, patient-related CINV risk factors were prospectively recorded, particularly among patients receiving MEC. These factors included female sex, age < 50 years, prior history of CINV, alcohol consumption < 5 times/week, motion sickness, pregnancy-related nausea, and pre-treatment anxiety or high nausea expectancy [21,22,23,24]. The total number of these individual risk factors was used to reflect cumulative patient-level risk burden.

Key exclusion criteria included nausea or vomiting unrelated to chemotherapy, recent use of antiemetic agents or radiotherapy, cognitive impairment, contraindications to corticosteroids, significant uncontrolled comorbidities, cardiac conduction abnormalities, drug hypersensitivity, pregnancy or lactation, recent investigational drug exposure, multi-day chemotherapy, and concomitant use of CYP3A4 modulators without appropriate washout (1 week for substrates/inhibitors; 4 weeks for inducers). Participants were discontinued for protocol-defined reasons, including serious adverse events (SAEs) where risks outweighed benefits, investigator-determined clinical necessity (e.g., disease progression), or voluntary withdrawal. All screening failures and early discontinuations were thoroughly documented with justification.

### 2.3. Randomization and Masking

Participants were randomly assigned in a 1:1 ratio to receive either 5 mg DEX (5D group) or 8 mg DEX (8D group) in combination with NEPA using a computer-generated randomization sequence (SPSS v26.0). Allocation was based on a predefined sequence of random values and implemented sequentially.

This study was conducted using an open-label design. Although blinding was considered during study planning, a double-blind design was not implemented due to differences in dosing and administration logistics, as well as the pragmatic nature of the trial. Importantly, the primary endpoint (complete response) was defined using objective criteria (absence of vomiting and no use of rescue medication), which are less susceptible to observer bias. Outcome data were collected using standardized patient diaries with predefined definitions to ensure consistency. Statistical analyses were performed according to a predefined analysis plan.

### 2.4. Procedures

Eligible patients received guideline-directed chemotherapy (including carboplatin, oxaliplatin, irinotecan, DS8201, Cisplatin, Nedaplatin, and liposomal doxorubicin combined with cyclophosphamide) dosed by body surface area (BSA) with renal/age adjustments. Thirty minutes pre-chemotherapy, the 5D group received intravenous 5 mg DEX followed by oral NEPA (300 mg netupitant/0.5 mg palonosetron) at 1 h before chemotherapy, while the 8D group received 8 mg DEX with identical NEPA timing.

Rescue antiemetic medications (including metoclopramide, olanzapine, or other standard agents) were permitted and pre-specified in the study protocol. According to the National Cancer Institute Common Terminology Criteria for Adverse Events version 5.0 (NCI-CTCAE v5.0), participants were instructed to use rescue medication in the event of any vomiting episode, moderate to severe nausea (grade ≥ 2), or symptoms interfering with daily activities. Importantly, rescue antiemetic therapy did not include study medications (dexamethasone or the 5-HT3 receptor antagonist component of NEPA), which were administered strictly according to the randomized study protocol and were not permitted as rescue treatment.

Patients were advised to contact the study team or treating physician if symptoms persisted or worsened, and guidance on rescue medication use was provided when necessary. The same instructions were given to all participants to ensure consistency across treatment groups. Use of any rescue medication during the specified observation period was considered treatment failure for the complete response endpoint, regardless of symptom severity.

Participants or caregivers recorded daily symptoms, including nausea, vomiting episodes, rescue medication use, and treatment-related adverse events (TRAEs) for 7 days following chemotherapy using standardized diaries with predefined categories. The observation period (0–168 h) was divided into three predefined phases: acute (0–24 h), delayed (24–120 h), and extended delayed (120–168 h). Outcome data were assessed daily, and for each phase, outcomes were summarized as whether events occurred at any time during the corresponding interval. To ensure data completeness and accuracy, clinical investigators conducted structured telephone follow-up assessments, using the NCI-CTCAE v5.0 to verify diary entries and evaluate symptom severity. When discrepancies occurred between diary records and telephone follow-up reports, the more conservative assessment (i.e., indicating the presence of symptoms or rescue medication use) was adopted to ensure consistency and avoid underestimation of events. All adverse events were closely monitored; SAEs required immediate intervention, mandatory reporting, and follow-up.

### 2.5. Outcomes

The primary endpoint was the complete response rate (CRR), defined as no vomiting and no use of rescue medication during the overall 0–168 h period following chemotherapy.

Secondary efficacy endpoints included phase-specific CRR during the acute (0–24 h), delayed (24–120 h), and long-delayed (120–168 h) phases, as well as safety outcomes. Safety assessments included cardiac toxicity monitoring via electrocardiograms (ECGs) performed at pre-dose and at 5 h after NEPA administration, along with blood glucose tracking (hyperglycemia defined as either fasting or postprandial glucose levels exceeding normal ranges) during the first week of treatment [25].

Exploratory efficacy endpoints included the total control (TC: no vomiting, rescue medications, or nausea) rate and complete control (CC: no vomiting/rescue medications, only grade 1 or less nausea) rate during acute, delayed, long-delayed, and overall post-chemotherapy phases, along with CINV incidence patterns and daily occurrence rates across these periods.

### 2.6. Statistical Analysis

The study was designed as a non-inferiority trial. Based on prior data, the expected CRR in both groups was assumed to be 80% [11,18,19]. The non-inferiority margin was set at −15%, consistent with previous CINV trials and determined at the design stage, considering both clinical relevance and feasibility [9,11,26].

Sample size calculations indicated that 176 patients (88 per group) would provide 80% power with a one-sided α of 0.05. To account for potential dropout, the target enrollment was increased to 186 patients.

Demographic and clinical characteristics were summarized using descriptive statistics, presented as frequencies (percentages) for categorical variables and median (interquartile range) for continuous variables. Group comparisons were performed using the chi-square or Fisher’s exact test for categorical variables, the Mann–Whitney U test for continuous variables between two groups, and the Kruskal–Wallis test for comparisons across multiple categories.

The primary endpoint was analyzed within a non-inferiority framework. The CRR during the overall 0–168 h period was calculated for each treatment group, and the between-group difference was defined as the response rate in the 5D group minus that in the 8D group. The prespecified non-inferiority margin was −15%. Non-inferiority was assessed using a one-sided Wald z test for the risk difference. The standard error of the risk difference was estimated using the unpooled binomial variance. Non-inferiority was considered demonstrated if the lower bound of the confidence interval for the risk difference exceeded the prespecified margin of −15%. Although the sample size was planned using a one-sided alpha level of 0.05, treatment differences are presented with two-sided 95% confidence intervals for conservative and transparent reporting.

For secondary and exploratory efficacy endpoints, response rates and between-group risk differences with corresponding 95% confidence intervals were summarized to assess consistency with the primary endpoint. These analyses were considered supportive and exploratory. Changes in response within subjects across different treatment phases were assessed using McNemar’s test. *p* values for the primary non-inferiority test were reported as one-sided, whereas other *p* values were two-sided.

A subgroup analysis of the primary efficacy outcome was performed, comparing the two dose cohorts using chi-square or Fisher’s exact tests. TRAEs were evaluated using Mann–Whitney U and chi-square tests.

Fasting glucose was measured at baseline. Post-chemotherapy hyperglycemia was assessed during the first week, typically on days 3–4. Elevation was defined as fasting glucose ≥6.1 mmol/L for non-diabetic patients and ≥7.0 mmol/L for those with known diabetes. For patients unable to fast due to advanced age or frailty, a 2 h postprandial glucose level was used as an alternative: ≥7.8 mmol/L for non-diabetic patients and ≥11.1 mmol/L for diabetic patients, per the World Health Organization 1998 diagnostic criteria [25].

Hyperglycemia adverse events were graded according to NCI-CTCAE v5.0. Grade 2 was defined as fasting glucose elevation requiring oral hypoglycemic agents, and grade 3 as glucose elevation requiring initiation of insulin therapy. All glucose measurements were recorded relative to DEX administration, and standardized monitoring protocols were followed.

Cardiotoxicity assessment via 12-lead ECG considered either more than 5% of patients developing corrected QT interval (QTcB, Bazett’s formula) exceeding 500 ms or over 15% showing QTcB prolongation greater than 60 ms from baseline [27,28]. When predefined QTcB thresholds were exceeded (absolute > 500 ms or increase from baseline > 60 ms), the treating physician was notified immediately. Serum electrolytes (potassium and magnesium) were measured and corrected if abnormal, and a repeat ECG was obtained within 2–4 h. Concomitant QT-prolonging medications were reviewed and discontinued if clinically appropriate. For persistent QTcB prolongation (>500 ms on two consecutive ECGs) or symptoms suggestive of arrhythmia (e.g., syncope, palpitations), a cardiology consultation was obtained. Further management, including temporary treatment interruption, was at the physician’s discretion. All ECG abnormalities were followed until resolution or stabilization, and outcomes were recorded for safety analysis.

Additional subgroup analyses of the primary endpoint were performed according to emetogenic risk category (HEC vs. MEC). Within each subgroup, CRRs were summarized descriptively, and risk ratios with corresponding 95% confidence intervals were estimated. Exploratory comparisons between treatment groups within subgroups were conducted using Fisher’s exact test. To further assess potential effect modification, an interaction analysis was performed by including an interaction term between treatment group and emetogenic risk in a logistic regression model. A two-sided *p* value < 0.05 for the interaction term was considered indicative of potential heterogeneity of treatment effect.

All statistical analyses used the modified intention-to-treat (mITT) population, except hyperglycemia and cardiotoxicity assessed in the per-protocol set (PPS) due to missing data. The mITT included eligible treated patients, excluding consent withdrawals or protocol violators, while PPS required full compliance and complete data records. Analyses were conducted using SPSS v26.0 and RStudio (version 4.3.3), with trial registration at ChiCTR2400089311.

### 2.7. Protocol Changes

No protocol changes occurred during the trial.

## 3. Results

### 3.1. Patient Disposition and Study Treatment Exposure

**Figure 1:**
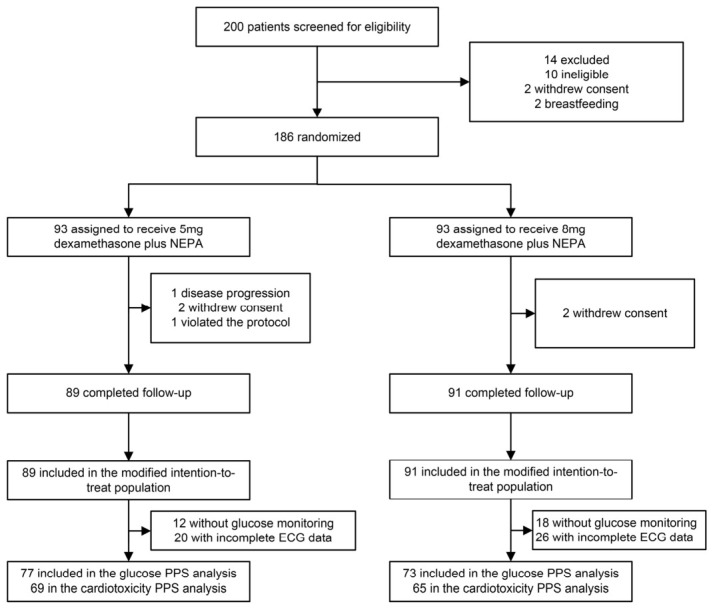
CONSORT Diagram. ECG, Electrocardiogram; PPS, per-protocol set.

A CONSORT flow diagram summarizing patient screening, randomization, follow-up, and analysis populations is presented in Figure 1. In this prospective trial, conducted between 1 October 2024 and 1 August 2025, a total of 200 patients were screened for eligibility. After excluding 14 patients (10 ineligible, 2 who withdrew consent, and 2 who were breastfeeding), 186 patients were randomized. Using computer-generated sequences, participants were equally assigned to the 5D and 8D treatment groups (*n* = 93 each). Six patients withdrew from the study, resulting in 180 patients who completed the modified intention-to-treat (mITT) analysis (89 in the 5D group and 91 in the 8D group). For safety analyses, patients with missing glucose or ECG data were excluded (Figure 1). The PPS for glucose analysis included 77 patients in the 5D group and 73 in the 8D group; the PPS for cardiotoxicity analysis included 69 patients in the 5D group and 65 in the 8D group.

### 3.2. Baseline Characteristics of Patients

Baseline demographics showed balanced characteristics between groups (Table 1), with median ages of 59.0 (5D, IQR 51.5–67.5) and 61 (8D, IQR 53.0–73.0) years, and male predominance (*n* = 108, 60.0%). All patients had an ECOG performance status of 0 or 1 and received HEC or MEC (with patient risk factors) regimens. The most frequent malignancies were colorectal, gastric, and lung cancers. While most parameters were comparable, the 8D group had a significantly higher proportion of patients with metastatic disease (78.0% vs. 62.9%, *p* = 0.026) and a lower proportion of elevated AST (14.3% vs. 27.0%, *p* = 0.035).

### 3.3. Efficacy Outcomes

The CRR during the overall phase was 78.7% in the 5D group compared to 70.3% in the 8D group. Statistical noninferiority of the 5D regimen versus the 8D regimen was demonstrated for the overall CRR, with a difference of 8.4% (95% CI: −1.5% to 19.0%; one-sided *p* < 0.0001). This conclusion was further supported by the fact that the lower bound of the 95% CI exceeded the predefined noninferiority margin of −15%. Results for secondary and exploratory efficacy endpoints were generally consistent with the primary analysis (Table 2).

### 3.4. Safety Outcomes

Overall, TRAEs were predominantly grade 1–2 and occurred with similar frequency between treatment groups.

Because dexamethasone and NEPA were administered concomitantly, adverse events could not be definitively attributed to a single agent. Therefore, safety analyses were interpreted in terms of overall treatment-emergent toxicity and a predefined set of steroid-associated adverse event patterns.

The most frequently reported adverse events included gastrointestinal symptoms (indigestion, constipation, and diarrhea), hiccups, insomnia, and transient hyperglycemia. Grade 3 events were rare, with only one case of grade 3 diarrhea reported in the 5 mg group. No treatment-related deaths occurred (Table S1).

Hyperglycemia was observed in 26.7% of patients in the 5 mg group and 35.6% in the 8 mg group (*p* = 0.81). Other steroid-associated adverse events, including insomnia, agitation, hiccups, and acneiform rash, showed no statistically significant differences between groups (Table S2).

Cardiac safety outcomes were comparable between groups. No clinically meaningful differences were observed in QTc prolongation (QTcB > 500 ms: *p* = 0.61; ΔQTcB > 60 ms: *p* = 0.43), and all abnormalities resolved spontaneously without sequelae.

Although a numerical reduction in several steroid-associated adverse events was observed in the 5 mg group, these differences did not reach statistical significance and should therefore be considered exploratory.

### 3.5. Exploratory Analysis

**Figure 2:**
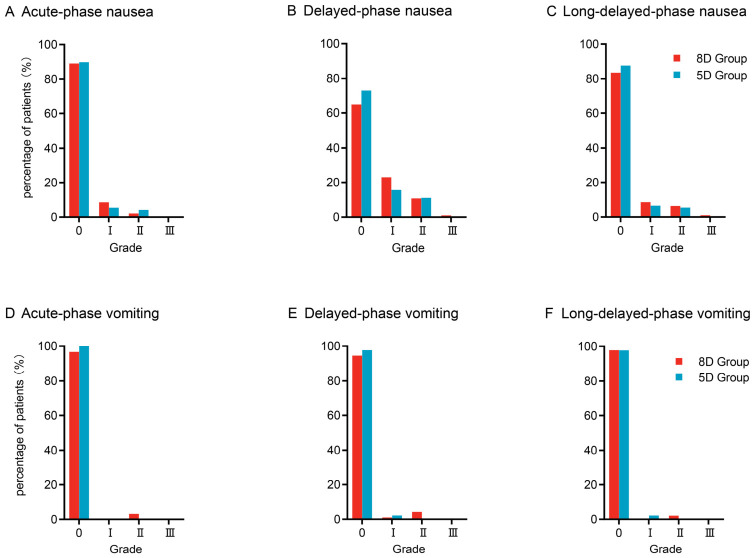
Incidence of nausea and vomiting at different stages post-chemotherapy. (**A**) Acute-phase nausea by grade. (**B**) Delayed-phase nausea by grade. (**C**) Long-delayed-phase nausea by grade. (**D**) Acute-phase vomiting by grade. (**E**) Delayed-phase vomiting by grade. (**F**) Long-delayed-phase vomiting by grade. Abbreviations: 5D, 5 mg dexamethasone combined with NEPA regimen; 8D, 8 mg dexamethasone combined with NEPA regimen.

Nausea/vomiting analysis revealed grade 1–2 events in both the 5D and 8D groups, predominantly peaking during the delayed phase (Figure 2). The daily incidence curves of CINV showed peak incidence rates on day 3 and day 2 post-chemotherapy, respectively (Figure S1). McNemar tests revealed significantly worse control of CINV during the delayed phase compared to other phases (Figure S2).

**Figure 3:**
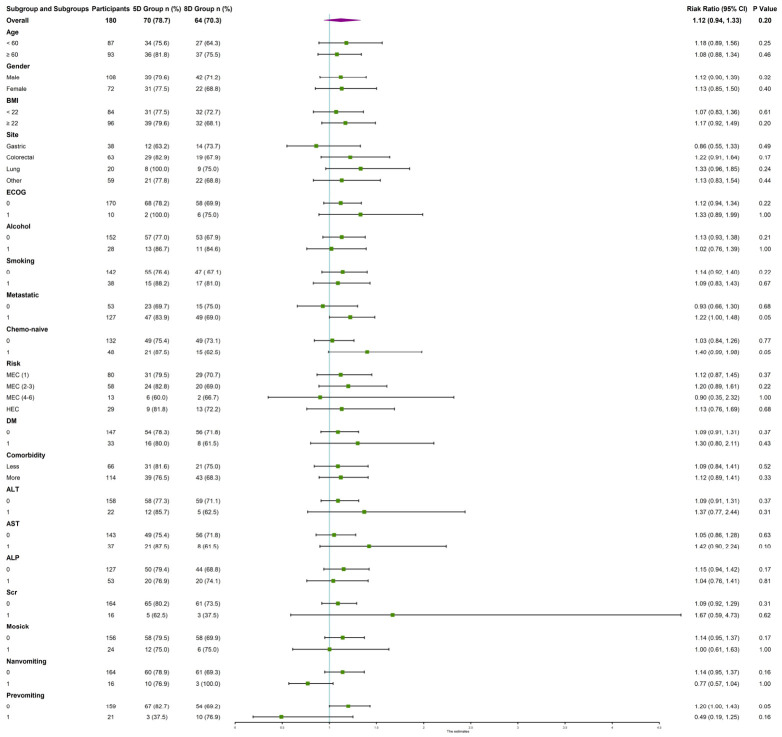
Subgroup Analysis. Risk ratios (RRs) with 95% confidence intervals are shown for each subgroup. The dashed vertical line at RR = 1 indicates no treatment difference. Squares represent the RR for each subgroup, horizontal lines represent the 95% CIs; the diamond indicates the overall estimate. Abbreviations: 5D, 5 mg dexamethasone plus NEPA regimen; 8D, 8 mg dexamethasone plus NEPA regimen; BMI, body mass index; ECOG, Eastern Cooperative Oncology Group score; MEC, moderately emetogenic chemotherapy; MEC (1), patients receiving MEC with 1 patient risk factor; MEC (2–3), patients receiving MEC with 2–3 patient risk factors; MEC (4–6), patients receiving MEC with patient 4–6 risk factors; HEC, highly emetogenic chemotherapy; DM, diabetes; ALT, alanine aminotransferase; AST, aspartate aminotransferase; ALP, alkaline phosphatase; Scr, serum creatinine; Mosick, motion sickness; Nanvomiting, morning sickness during pregnancy; Prevomiting, prior chemotherapy-induced nausea and vomiting history. Comorbidity burden was categorized as less (0–4 clinically significant comorbid conditions) or more (>4 clinically significant comorbid conditions) based on baseline medical history. *p* values less than 0.05 are considered statistically significant.

Furthermore, we conducted a pre-specified subgroup analysis of the CRR during the overall phase (0–168 h) to assess the consistency of the treatment effect across various baseline characteristics (Figure 3). The subgroup variables were selected based on established or commonly reported patient-related risk factors for CINV, including age, sex, prior CINV, history of pregnancy-related vomiting, alcohol consumption, disease characteristics, and treatment-related factors. Comorbidity burden was assessed according to the presence of clinically relevant chronic conditions, including hypertension, diabetes mellitus, cardiovascular disease, chronic pulmonary disease, and other documented medical disorders. Patients were categorized as having fewer comorbidities (0–4 conditions) or more comorbidities (>4 conditions) to reflect overall disease burden [29]. No statistically significant treatment-by-subgroup interactions were observed, although trends indicated a possible increased risk associated with 5D in patients with gastric cancer, non-metastatic, multiple emetogenic risk factors, prior CINV, or a history of pregnancy-related vomiting.

Additionally, the subgroup analysis by the number of individual emetogenic risk factors (Figure 3) suggested a potential numerical trend in patients with the highest risk burden. While the risk ratios (5D vs. 8D) for patients with 1 factor (1.12; 95% CI: 0.87–1.45) and 2–3 factors (1.20; 95% CI: 0.89–1.61) were above 1.0, the point estimate in the subgroup of patients with 4–6 factors was 0.90 (95% CI: 0.35–2.32). This observation may suggest a possible reduction in efficacy of the 5 mg regimen in patients with multiple emetogenic risk factors. However, this subgroup included a very limited number of patients (*n* = 13), and the confidence interval was wide and crossed unity, indicating substantial statistical uncertainty. Therefore, this finding should be interpreted with caution and cannot be considered conclusive.

To address the potential heterogeneity between HEC and MEC, we conducted a prespecified stratified analysis of the primary endpoint. The CRR remained comparable between the 5 mg and 8 mg DEX groups within both HEC and MEC subgroups (Table S3). We further performed a formal test for interaction to determine whether the treatment effect varied according to emetogenic risk category. No statistically significant interaction was observed (*p* for interaction = 0.912), indicating no evidence that the relative treatment effect of the 5 mg regimen compared with the 8 mg regimen was modified by emetogenic risk.

## 4. Discussion

This randomized phase III trial demonstrates that a reduced single dose of DEX (5 mg), when combined with NEPA, is non-inferior to the conventional 8 mg dose for the prevention of CINV over 168 h in patients receiving MEC or HEC. The observed treatment effect did not approach the predefined non-inferiority margin, and the point estimate numerically favored the lower-dose regimen, suggesting that a clinically meaningful loss of efficacy is unlikely. Consistent findings across phase-specific and exploratory endpoints further support the robustness of this result.

The numerically higher complete response rate observed in the 5 mg group should be interpreted carefully. The study was not powered for superiority testing, and the confidence interval included the possibility of no true difference. This observation may reflect random variation rather than a true treatment advantage. Accordingly, our conclusions remain focused on non-inferiority rather than superiority.

These findings should be interpreted within the broader effort to optimize antiemetic regimens by reducing unnecessary corticosteroid exposure. DEX remains a key component of guideline-recommended therapy; however, its use is associated with dose-dependent adverse effects, including metabolic, neuropsychiatric, and immunologic complications [8,30,31].

Given that antiemetic prophylaxis is administered repeatedly across chemotherapy cycles, even modest reductions in per-cycle DEX exposure may translate into clinically meaningful cumulative benefits, particularly in patients with underlying comorbidities or increased susceptibility to steroid-related toxicity.

In this context, the present study was designed as a stepwise dose-reduction strategy rather than a withdrawal approach. Although further reduction in DEX exposure may be conceptually appealing, existing evidence suggests that complete omission may not be appropriate in all clinical settings. Prior randomized studies evaluating DEX-sparing strategies in NK1 RA–based regimens have reported variable efficacy, particularly in the delayed phase of CINV. Moreover, current international guidelines continue to recommend DEX as a backbone component of antiemetic therapy, especially for patients receiving HEC, where optimal control of delayed symptoms remains critical. Accordingly, identifying the lowest effective dose—rather than testing omission—represents a more pragmatic and clinically applicable approach.

The selection of 5 mg was based on a clinically acceptable intermediate reduction that is both practical for routine administration. Whether even lower doses or complete omission can provide comparable protection against CINV remains an important question for future investigation.

In the present study, no statistically significant differences in TRAEs were observed between groups, although events occurred numerically less frequently in the reduced-dose group. However, because DEX and NEPA were administered as a fixed combination, adverse events could not be reliably attributed to either agent alone. Therefore, we interpreted safety outcomes as overall treatment-emergent effects, with a specific focus on steroid-associated patterns, including hyperglycemia, insomnia, agitation, hiccups, and acneiform rash. Although the study was not powered to detect safety differences, the observed numerical trends are directionally consistent with the hypothesis that reduced corticosteroid exposure may improve tolerability. These findings should be interpreted cautiously, as they are exploratory and hypothesis-generating rather than confirmatory; nonetheless, they provide supportive evidence for the feasibility of dose reduction.

Subgroup analyses showed generally consistent treatment effects across baseline characteristics. A numerical trend toward reduced efficacy was observed in patients with the highest number of emetogenic risk factors; however, this finding was based on a small sample size and was associated with wide confidence intervals, indicating substantial uncertainty. Importantly, no statistically significant interaction between treatment effect and emetogenic risk category was identified, suggesting no clear evidence of effect modification. Nevertheless, caution may be warranted when applying reduced-dose strategies in patients with a high burden of risk factors.

The choice of a 15% non-inferiority margin and a one-sided alpha of 0.05 may be considered relatively permissive. However, these parameters were prespecified at the design stage and jointly determined with the anticipated response rate and feasible sample size. Importantly, the observed treatment effect remained well within the non-inferiority boundary and did not approach the margin, indicating that the conclusions are unlikely to be sensitive to these assumptions.

The control rates observed in the delayed phase (24–120 h) were lower than those in both the acute and long-delayed phases. This pattern may, in part, reflect the pharmacodynamic characteristics of NEPA. Previous imaging studies have shown that NK1 receptor occupancy in the central nervous system gradually declines over time following a single dose of netupitant, which may contribute to relatively reduced antiemetic control during the delayed phase [32].

Notably, nausea remained substantially more frequent than vomiting in the present study, particularly during the delayed phase. This discrepancy may suggest partially distinct neuroregulatory mechanisms underlying nausea and vomiting. It also highlights the persistent challenge of delayed nausea control despite effective suppression of emesis. Together, these findings underscore the importance of optimizing antiemetic strategies specifically for the delayed phase. While the present study was not designed to evaluate alternative dosing schedules, emerging evidence suggests that modified or repeated dosing strategies may warrant further investigation in selected clinical settings, particularly for patients with persistent or refractory symptoms [33].

Beyond the primary efficacy findings, this study provides additional clinical insights relevant to the optimization of antiemetic prophylaxis. A simplified regimen consisting of single-dose NEPA combined with a reduced dose of DEX demonstrated sustained efficacy over the full 168 h observation period, covering acute, delayed, and extended delayed phases. This extended monitoring offers a more comprehensive assessment of delayed-phase control compared with conventional 120 h endpoints. Such a regimen may be particularly relevant for patients in whom corticosteroid exposure is a concern, including those with metabolic comorbidities or prior steroid-related adverse effects. These findings support the feasibility of treatment simplification while maintaining clinical effectiveness.

Several limitations of this study should be acknowledged. First, this was a single-center study, which may limit the generalizability of the findings. Patient characteristics, chemotherapy regimens, antiemetic practice patterns, and institutional approaches to corticosteroid dose reduction may differ across regions. Multicenter studies including more diverse populations and practice settings are needed to confirm the external applicability of these findings. Second, the sample size was not designed to support definitive conclusions within specific subgroups, including patients receiving HEC or those with multiple emetogenic risk factors. Third, we acknowledge that the selected non-inferiority margin may be considered relatively permissive; the absence of a formal effect preservation analysis represents a statistical limitation and should be taken into account when interpreting the primary endpoint. Fourth, the open-label design may have introduced potential bias, particularly in the assessment of subjective symptoms such as nausea. However, the primary endpoint included objective components, such as the absence of vomiting and no use of rescue medication, which may reduce observer-related bias.

In addition, the study was not powered to detect differences in safety outcomes or longer-term cumulative corticosteroid toxicity across multiple chemotherapy cycles. Importantly, corticosteroid-related toxicities were not comprehensively or prospectively captured in this study. While commonly reported events such as hyperglycemia, insomnia, and gastrointestinal symptoms were monitored, other clinically relevant effects—including mood changes, appetite alterations, infection risk, and broader quality-of-life outcomes—were not systematically assessed. This limitation highlights the need for future studies incorporating standardized patient-reported outcome measures and more detailed toxicity profiling to better characterize the clinical benefits and trade-offs of corticosteroid dose reduction. Finally, the absence of a conventional 12 mg DEX comparator limits direct comparison with some guideline-based antiemetic regimens, although the 8 mg regimen was selected based on prior institutional experience and emerging evidence supporting dexamethasone-sparing strategies.

## 5. Conclusions

This study demonstrates that a single low dose of 5 mg DEX combined with NEPA is non-inferior to an 8 mg regimen in preventing CINV over 168 h, with comparable safety. This simplified regimen reduces corticosteroid exposure and may support treatment optimization in clinical practice.

## Figures and Tables

**Table 1:** Baseline characteristics.

Characteristics	No. (%) of Participants		
	5D Group (*n* = 89)	8D Group (*n* = 91)	*p*-Value ^a^
Age, median (range), years	59.0 (51.5–67.5)	61.0 (53.0–73.0)	0.376
Gender			
Male	49 (55.1)	59 (64.8)	0.181
Female	40 (44.9)	32 (35.2)	
BMI, median (range), kg/m^2^	22.5 (19.9–24.8)	22.2 (19.8–24.4)	0.850
Site			
Gastric	19 (21.3)	19 (20.9)	0.427
Colorectal	35 (39.3)	28 (30.8)	
Lung	8 (9.0)	12 (13.2)	
Other	27 (30.3)	32 (35.2)	
ECOG performance status			
0	87 (97.8)	83 (91.2)	0.100
1	2 (2.2)	8 (8.8)	
Alcohol ^b^			
No	74 (83.1)	78 (85.7)	0.635
Yes	15 (16.9)	13 (14.3)	
Smoking ^b^			
No	72 (80.9)	70 (76.9)	0.513
Yes	17 (19.1)	21 (23.1)	
Metastatic			
No	33 (37.1)	20 (22.0)	0.026
Yes	56 (62.9)	71 (78.0)	
Chemotherapy naive			
No	65 (73.0)	67 (73.6)	0.928
Yes	24 (27.0)	24 (26.4)	
Risk			
MEC (1)	39 (43.8)	41 (45.1)	0.139
MEC (2–3)	29 (32.6)	29 (31.9)	
MEC (4–6)	10 (11.2)	3 (3.3)	
HEC	11 (12.4)	18 (19.8)	
Diabetes			
No	69 (77.5)	78 (85.7)	0.156
Yes	20 (22.5)	13 (14.3)	
ALT			
Normal	75 (84.3)	83 (91.2)	0.155
Elevated	14 (15.7)	8 (8.8)	
AST			
Normal	65 (73.0)	78 (85.7)	0.035
Elevated	24 (27.0)	13 (14.3)	
ALP			
Normal	63 (70.8)	64 (70.3)	0.946
Elevated	26 (29.2)	27 (29.7)	
Scr			
Normal	81 (91.0)	83 (91.2)	0.963
Elevated	8 (9.0)	8 (8.8)	

Notes: 5D, 5 mg dexamethasone plus NEPA; 8D, 8 mg dexamethasone plus NEPA; BMI, body mass index (calculated as weight in kilograms divided by height in meters squared), ECOG Eastern Cooperative Oncology Group score; MEC (1), moderately emetogenic chemotherapy with 1 risk factor; MEC (2–3), 2–3 risk factors; MEC (4–6), 4–6 risk factors; HEC, highly emetogenic chemotherapy; ALT, alanine aminotransferase; AST, aspartate aminotransferase; ALP, alkaline phosphatase, Scr serum creatinine. a: *p* < 0.05 was considered statistically significant (two-sided). b: Current smokers reported smoking within 6 months; drinkers consumed alcohol ≥5 times/week.

**Table 2:** Efficacy endpoints.

Outcomes	Phase	5D Group (*n* = 89)	8D Group (*n* = 91)	Difference (95% CI)	*p*-Value *
CRR	Acute	84 (94.4%)	85 (93.4%)	1.0% (−5.1–7.2%)	-
	Delayed	70 (78.7%)	64 (70.3%)	8.4% (−1.5–19.0%)	-
	Long-delayed	81 (91.0%)	76 (83.5%)	7.5% (−0.3–15.7%)	-
	Overall	70 (78.7%)	64 (70.3%)	8.4% (−1.5–19.0%)	<0.0001
TCR	Acute	80 (89.9%)	79 (86.8%)	3.1% (−4.7–11.1%)	-
	Delayed	64 (71.9%)	58 (63.7%)	8.2% (−2.7–19.8%)	-
	Long-delayed	78 (87.6%)	76 (83.5%)	4.1% (−4.2–12.9%)	-
	Overall	64 (71.9%)	58 (63.7%)	8.2% (−2.7–19.8%)	-
CCR	Acute	85 (95.5%)	87 (95.6%)	−0.1% (−5.2–5.2%)	-
	Delayed	77 (86.5%)	78 (85.7%)	0.8% (−7.4–9.6%)	-
	Long-delayed	84 (94.4%)	83 (91.2%)	3.2% (−3.1–9.8%)	-
	Overall	77 (86.5%)	78 (85.7%)	0.8% (−7.4–9.6%)	-

Notes: 5D, 5 mg dexamethasone plus NEPA; 8D, 8 mg dexamethasone plus NEPA; CRR, complete response rate; TCR, total control rate; CCR, complete control rate. *: The primary endpoint (overall CRR 0–168 h) was analyzed using a one-sided non-inferiority test with the prespecified margin of −15%; the associated *p* value reflects the non-inferiority hypothesis. Secondary and exploratory endpoints (TC, CC, phase-specific CR) are presented descriptively with risk differences and 95% confidence intervals and are not associated with formal non-inferiority testing. *p* < 0.05 was considered statistically significant.

## Data Availability

The data underlying this article are available from the corresponding author (provide email: xlshb0826@xmu.edu.cn) upon reasonable request.

## Supplementary Materials

The following supporting information can be downloaded at: https://www.mdpi.com/article/10.3390/diseases14070231/s1, including the CONSORT 2025 checklist, the full trial protocol and the SPIRIT checklist. Table S1: Treatment-Related Adverse Events; Table S2: Steroid-associated adverse events during treatment; Table S3: Complete response rates stratified by emetogenic risk category (HEC vs. MEC). Figure S1: Daily incidence of nausea and vomiting. (A) Daily incidence of nausea. (B) Daily incidence of vomiting; Figure S2: The temporal trends of various outcome endpoints. (A) Phase-Specific CR. (B) Phase-Specific TC. (C) Phase-Specific CC. Abbreviations: 5D, 5 mg dexamethasone combined with NEPA regimen; 8D, 8 mg dexamethasone combined with NEPA regimen; CR, complete response; TC, total control; CC, complete control. The McNemar test compared differences in three endpoints (CR, TC, CC) across treatment stages. * *p* < 0.05, ** *p* < 0.01, *** *p* < 0.001.

## Abbreviations

ALT	Alanine Aminotransferase
ASCO	American Society of Clinical Oncology
AST	Aspartate Aminotransferase
BSA	Body Surface Area
CCR	Complete Control Rate
CI	Confidence Interval
CINV	Chemotherapy-induced Nausea and Vomiting
CRR	the complete response rate
CTCAE	the Common Terminology Criteria for Adverse Events
DEX	Dexamethasone
ECG	Electrocardiogram
ECOG	Eastern Cooperative Oncology Group
ESMO	European Society for Medical Oncology
HEC	Highly Emetogenic Chemotherapy
MASCC	Multinational Association of Supportive Care in Cancer
MEC	Moderately Emetogenic Chemotherapy
mITT	the modified intention-to-treat population
NCCN	National Comprehensive Cancer Network
NEPA	Netupitant-Palonoseton
NK-1 RA	NK-1 receptor antagonist
PPS	Per-protocol Set
QTcB	QT interval corrected by Bazett’s formula
RCT	Randomized Controlled Trial
SAE	Serious Adverse Event
TCR	Total Control Rate
TRAE	Treatment-Related Adverse Event
5-HT3	5-hydroxytryptamine-3
