# Supplementary material for: Phase III Trial: Single Low-Dose 5 mg Dexamethasone with NEPA for Preventing 168 h Nausea and Vomiting of Diverse Highly or Moderately Emetogenic Chemotherapy (LD-NEPA)
Source: Diseases. 2026 Jun 27;14(7):231. doi: 10.3390/diseases14070231 (PMC13407607; doi:10.3390/diseases14070231)
Supplement: Supplementary file 1 [file diseases-14-00231-s001.zip › Protocol.pdf]

Single Low-Dose Dexamethasone（5mg Versus 8mg） With NEPA For Prevention Of Highly Or Moderately Emetogenic Chemotherapy (LD-NEPA): A Phase III Randomised Clinical Trial

Protocol No.: 2024-102
Study Phase: Phase III
Drug Name: Dexamethasone for injection and oral NEPA
Protocol Owner: Li Xiao
Study Leading Site: Xiamen University Affiliated Zhongshan Hospital
Principal Investigator: Professor Xiao Li

Version No.: V2.0
Version Date: June 10, 2024

Sponsor: Xiamen University Affiliated Zhongshan Hospital
Department of Oncology
No. 22 Lujiang Road, Siming District, Xiamen, Fujian, China 361004

Confidentiality Statement

The information contained in this protocol is confidential and is intended for use by clinical investigators only. It must not be disclosed except as required by applicable laws or regulations. The copyright of this protocol is owned by Xiamen University Affiliated Zhongshan Hospital and must not be copied or distributed to any person who has not participated in this clinical study, except for those who have signed a confidentiality agreement with Xiamen University Affiliated Zhongshan Hospital.

VERSION HISTORY/AMENDMENT HISTORY

Version No.	Version Date	Explanation of the rationale for amendment and summary of changes
1.0	May 15, 2024	Not applicable
2.0	June 10, 2024	1: In the informed consent form, the responsibility for adverse events is supplemented as follows: Although this study is conducted with the premise of ensuring the safety of participants, there is still a possibility of unforeseeable serious adverse reactions that may lead to health damage. If a participant experiences an unforeseeable serious adverse reaction related to the study protocol that results in health damage, the responsible physician will conduct active diagnosis and treatment. In the event that a participant suffers harm that is determined by an authoritative institution as stipulated by national laws and regulations to be related to the investigational drug of this study and for which responsibility is to be assumed, the participant will receive free treatment and compensation in accordance with the laws and regulations of China. The investigator (physician) will do their utmost to prevent and treat any harm that may arise from this study.<br>2: The protocol has been supplemented to clarify the risks that the experimental group will

		undertake, and both the protocol and the informed consent form have been revised accordingly. The content is as follows: This study aims to explore the possibility of reducing the use of glucocorticoids on the basis of standard treatment, with the expectation of effectively preventing chemotherapy-induced nausea and vomiting (CINV) while reducing the types, incidence, and severity of potential adverse reactions. Although this approach may decrease the incidence and severity of side effects associated with dexamethasone (DEX), the reduction in DEX dosage may increase the risk of poor CINV control in the experimental group. This could potentially lead to decreased adherence to chemotherapy and a reduced quality of life.<br><br>3: The protocol has been supplemented to include screening for cardiotoxicity. Specifically, electrocardiogram (ECG) tests will be conducted at three distinct time points: during the screening period, before antiemetic treatment on the day of chemotherapy, and 5 hours after chemotherapy.<br><br>4: The informed consent form has been supplemented to clarify the potential benefits for participants. The potential benefits for subjects in this study are primarily reflected in the reduction of nausea and vomiting caused by

		chemotherapy through a more convenient and sustained method. This, in turn, is expected to further enhance the quality of life, avoid reducing chemotherapy doses, delaying chemotherapy, or even suspending chemotherapy. Indirect benefits include the mitigation of adverse reactions associated with antiemetic medications, such as insomnia, gastrointestinal symptoms, anxiety and irritability, rashes, and increased risk of infection. Additionally, completing chemotherapy at full doses, for the full course, and on schedule will also improve the treatment outcomes and prognosis for cancer patients.

Protocol Signature Page

I, as the physician participating in this study, have read the protocol of this research.

I have fully discussed the purpose of this study and the content of this protocol with the study leader.

I agree to conduct the study in accordance with this protocol, comply with its requirements, adhere to ethical standards, and carry out this clinical study under the guidance of Good Clinical Practice (GCP).

I agree to keep the content of this protocol confidential and not to disclose it to any third party, and the content of the protocol is to be used solely for the conduct of this study.

I understand that if a decision is made to suspend or terminate this study at any time for any reason, the study leader will notify me in writing. Similarly, if I decide to withdraw from conducting this study, I will immediately notify the lead unit and the principal investigator of this study in writing.

Signature: ________________

Date: __________________

Table of Contents

Protocol Signature Page **5**
Table of Contents **6**
Abbreviations **9**
Protocol Synopsis **10**
Study Flowchart **18**
1. Research Background and Rationale **21**
1.1 Chemotherapy-Induced Nausea and Vomiting (CINV) 21
1.2 Current Treatment and Research Status of CINV 22
1.3 Potential Risks and Benefits 23
1.3.1 Known Potential Risks 23
1.3.2 Known Potential Benefits 26
2. Research Objectives and Endpoint Goals **26**
2.1 Research Objectives 26
2.1.1 Primary Objective 26
2.1.2 Secondary Objectives 26
2.2 Research Indicators 26
2.2.1 Primary Endpoint 26
2.2.2 Secondary Endpoints 27
2.2.3 Safety Endpoints 27
3. Study Design **27**
3.1 Screening Period 27
3.2 Pre-treatment Period 27
4. Selection and Withdrawal of Study Subjects **28**
4.1 Inclusion Criteria 28
4.2 Exclusion Criteria 29
4.3 Withdrawal Criteria 30
5. Study Medications **30**
5.1 Basic Information of Medications 30
5.2 Dosing Regimen 31
5.2.1 Drug Dose 31
5.2.2 Drug Discontinuation Criteria During Treatment 31
5.3 Concomitant Medication 31
5.3.1 Permitted Medications Prior to Study 32
5.3.2 Permitted Medications During the Study 32
5.3.3 Prohibited or Cautiously Used Medications and Treatments During the Study . 32
5.4 Medication Adherence 32
6 Study Procedures **32**
6.1 Screening Phase 33
6.2 Treatment Phase 33
6.3 Follow-up Phase 34

6.3.1 End of Study Treatment/Withdrawal Visit..34
6.3.2 Safety Follow-up Visit..34
7 Efficacy Evaluation..34
7.1 Primary Efficacy Endpoint...34
7.2 Secondary Efficacy Endpoints...35
8 Safety Evaluation..35
8.1 Physical Examination and Vital Signs...36
8.2 Laboratory Tests...36
8.3 Nausea and Vomiting Assessment..36
8.4 Adverse Events (AEs)..37
8.4.1 Definition of Adverse Events...37
8.4.2 Criteria for Assessing the Severity of Adverse Events...37
8.4.3 Criteria for Assessing the Relationship Between Adverse Events and the Study Drug..........38
8.4.4 Recording and Reporting of Adverse Events..39
8.5 Serious Adverse Events (SAEs)...40
8.5.1 Definition of Serious Adverse Events..40
8.5.2 Tumor Disease Progression..40
8.6 Research-Related Injury and Compensation...40
9 Management of the Research Project...41
9.1 Ethical Standards and Informed Consent..41
9.1.1 Ethical Standards..41
9.1.2 Informed Consent...41
9.3 Drug Management...42
9.4 Protocol Amendments...42
9.5 Monitoring..42
9.6 Quality Control and Assurance...42
9.7 Data Management..43
9.7.1 Data Collection...43
9.7.2 Data Management and Quality Control..43
9.7.3 Data Review...43
9.8 Protocol Deviations...44
9.9 Publication of Study Results...44
10 Data Analysis and Statistical Methods...44
10.1 Sample Size Estimation...44
10.2 Randomization and masking..44
10.3 Analysis Populations..45
10.4 Handling of Missing Data..45
10.5 General Principles for Efficacy Analysis...45
10.6 General Principles for Safety Analysis..46
11 Dropout of Subjects...46
12 Study Timeline...47
References..48
Appendix 1: ECOG Performance Status Scale (Eastern Cooperative Oncology

Group)......51
Appendix 2: NCCN Emetic Risk Classification for Anticancer Agents......52
Appendix 3: Serious Adverse Event (SAE) Report Form (Standardized template based on ICH-GCP guidelines)......53
Appendix 4: NCI CTCAE 5.0......55

Abbreviations

- DEX Dexamethasone
- NEPA Netupitant/Palonosetron Capsules
- CINV Chemotherapy-Induced Nausea and Vomiting
- CRR Complete Response Rate
- NSNR No Significant Nausea Rate
- CPR Complete Protection Rate
- mITT Modified Intention-To-Treat
- PPS Per Protocol Set
- ASCO American Society of Clinical Oncology
- NCCN National Comprehensive Cancer Network
- MASCC Multinational Association of Supportive Care in Cancer
- ESMO European Society for Medical Oncology
- MEC Moderately Emetogenic Chemotherapy
- HEC Highly Emetogenic Chemotherapy
- NK1RA Neurokinin-1 Receptor Antagonist
- 5-HT3RA 5-Hydroxytryptamine-3 Receptor Antagonist

Protocol Synopsis

**Study Title**	Single Low-Dose Dexamethasone（5mg Versus 8mg）With NEPA For Prevention Of Highly Or Moderately Emetogenic Chemotherapy (LD-NEPA): A Phase III Randomised Clinical Trial
**Protocol Number**	2024-102
**Version Number**	V2.0
**Sponsor**	Xiamen University Affiliated Zhongshan Hospital
**Principal Investigator**	Dr. Xiao Li
**Nature of the Study**	Investigator-Initiated Study
**Study Design**	Prospective, Randomized, Non-inferiority Trial
**Study Population**	Patients with chemotherapy-induced nausea and vomiting (CINV) due to malignant tumors
**Research Question**	To explore the clinical efficacy of different low-dose dexamethasone combined with single or double standard doses of netupitant/palonosetron capsules (NEPA) for the prevention of CINV.
**Study Objectives**	Primary Objective:<br>To explore the efficacy of different low-dose dexamethasone combined with single dose of NEPA in preventing CINV.<br>Secondary Objectives:<br>To evaluate the safety of different low-dose dexamethasone combined with single dose of NEPA in treating CINV.

**Study Endpoints**	Primary Endpoint:<br>Complete Response Rate (CRR, defined as no vomiting episodes and no rescue medication use within 7 days after receiving the study drug).<br>Secondary efficacy Endpoints:<br>• CRR during acute (0-24h), delayed (24-120h), and long-delayed (120-168h) phases<br>Secondary Safety Endpoints:<br>• Treatment related adverse events (TRAEs, evaluated using the National Cancer Institute Common Terminology Criteria for Adverse Events (NCI CTCAE) version 5.0).<br>• Cardiac toxicity monitoring via pre-dose and 5-hour post-dose electrocardiograms (ECGs)<br>• Blood glucose tracking (hyperglycemia defined as fasting/postprandial glucose exceeding normal ranges) during the first treatment week.<br>Exploratory endpoints:<br>• total control rate (TCR: no vomiting, rescue medications, or nausea) and complete control rate (CCR: no vomiting/rescue medications, only grade 1 or less nausea) during acute, delayed, long-delayed, and overall post-chemotherapy phases.<br>• CINV incidence patterns and daily occurrence rates across each period.

**Inclusion Criteria**	Patients must meet all of the following criteria to be eligible for the study:<br>1. Age 18-75 years.<br>2. Diagnosed with malignant tumors and receiving chemotherapy with moderate to high emetic risk.<br>3. ECOG performance status 0-1.<br>4. Expected survival ≥12 weeks at screening and able to receive at least one cycle of the current chemotherapy regimen.<br>5. Adequate organ function meeting the following criteria:<br>a: Neutrophil count ≥ 1.5 × 109/L;<br>b: Platelet count ≥ 100×109/L;<br>c: In patients without known liver metastases, aspartate aminotransferase ≤ 3 × ULN and/or alanine aminotransferase ≤ 3 × ULN (for patients with liver metastases, it can be relaxed to ≤ 5 × ULN);<br>6. Fertile subjects agree to use reliable contraception (e.g., condoms, contraceptive foam, IUD) throughout the study. Postmenopausal women (>1 year) and subjects with bilateral oophorectomy, bilateral vasectomy, or bilateral tubal ligation are exempt.<br>7. Voluntarily participating in the study with signed informed consent and good compliance.
**Exclusion Criteria**	Patients with any of the following conditions are not eligible for the study:

	1. Received radiotherapy within 1 week before chemotherapy.<br>2. Other diseases causing nausea or vomiting, such as gastrointestinal obstruction or central nervous system malignancies.<br>3. Vomiting, nausea, or mild nausea symptoms within 24 hours before treatment.<br>4. Concurrent use of corticosteroids or any other antiemetic drugs.<br>5. Contraindications to corticosteroids.<br>6. Cognitive impairment (e.g., dementia or severe learning difficulties) that prevents completion of nausea/vomiting assessment scales.<br>7. Use of antipsychotic drugs within 30 days before or during treatment.<br>8. Severe uncontrolled diseases affecting the liver, kidneys, cardiovascular system, respiratory system, endocrine system, or central nervous system.<br>9. Allergy to study drugs.<br>10. Pregnant or breastfeeding women.<br>11. Participation in another clinical study involving investigational drugs or devices within 3 months before screening.

	12. Any other condition that the investigator deems a significant risk to the subject's health or safety or that may affect efficacy assessment.
**Withdrawal Criteria**	Patients will be withdrawn from the study if any of the following occurs:<br>1. Withdrawal of informed consent or request to exit the study and refusal of further follow-up.<br>2. Investigator deems it necessary to withdraw the patient (e.g., loss of freedom to express willingness due to imprisonment or quarantine).<br>3. Loss to follow-up.<br>4. Serious adverse events (SAEs) where risks outweighed benefits<br>5. Termination of the study by the sponsor, investigator, or regulatory authority.
**Study Design**	This study is a randomized, non-inferiority trial with control and experimental groups, divided into screening and treatment phases.<br>1. Screening Phase<br>• Assess eligibility criteria. Eligible patients proceed to the treatment phase.<br>2. Treatment Phase<br>• Control Group: Intravenous injection of 8 mg dexamethasone 0.5 hours before chemotherapy and oral administration of single dose of NEPA (each containing 0.3 g netupitant and 0.5 mg palonosetron hydrochloride) 1 hour before chemotherapy.

	• Experimental Group: Intravenous injection of 5 mg dexamethasone 0.5 hours before chemotherapy and oral administration of single dose of NEPA (each containing 0.3 g netupitant and 0.5 mg palonosetron hydrochloride) 1 hour before chemotherapy.<br>Stratification Factors: Gender, age, CINV risk factors, ECOG score, tumor type, distant metastasis of the tumor, emetic risk of the chemotherapy regimen, smoking history, alcohol consumption history, etc.<br>Discontinuation Criteria: Discontinue treatment if the patient experiences severe adverse events or specific types of adverse events, such as severe drug intolerance or allergic reactions.
**Study Drugs**	• Dexamethasone Sodium Phosphate Injection<br>• Netupitant/Palonosetron Capsules (each containing 0.3 g netupitant and 0.5 mg palonosetron hydrochloride)
**Dosing Schedule**	Control Group: Intravenous injection of 5 mg dexamethasone 0.5 hours before chemotherapy and oral administration of 1 capsule of netupitant/palonosetron (each containing 0.3 g netupitant and 0.5 mg palonosetron hydrochloride) 1 hour before chemotherapy.<br>Experimental Group: Intravenous injection of 8 mg dexamethasone 0.5 hours before chemotherapy and oral administration of 1 capsule of netupitant/palonosetron (each containing 0.3 g netupitant and 0.5 mg palonosetron hydrochloride) 1 hour before chemotherapy.

	Discontinuation Criteria: Discontinue treatment if the patient experiences severe adverse events or specific types of adverse events, such as severe drug intolerance or allergic reactions.
**Data Collection**	Data related to the study treatment will be recorded within 7 days after the patient completes chemotherapy. Past medical history data will be collected based on medical records and patient interviews and entered into an electronic data capture (EDC) system.
**Sample Size Calculation**	186 subjects
**Data Analysis/Statistical Methods**	• Intention-To-Treat (ITT): Includes all randomized subjects, regardless of whether they completed the study, deviated from the protocol, or dropped out.<br>• Modified Intention-To-Treat (mITT): mITT is a variation of ITT that excludes certain participants post-randomization based on predefined criteria (e.g., major protocol violations, ineligibility, or lack of any post-baseline data).<br>• Per Protocol Set (PPS): includes only participants who fully adhered to the trial protocol (e.g., completed treatment, complied with visits, and had no major violations).<br>• All statistical analyses will be performed using SPSS 26.0 statistical software and RStudio v4.3.3. All statistical tests will be two-sided, with a significance level of $P \leq 0.05$.

**Study Timeline**	• First subject enrollment: October 2024<br>• Estimated last subject enrollment: August 2025<br>• Estimated study completion date: August 2025

Study Flowchart

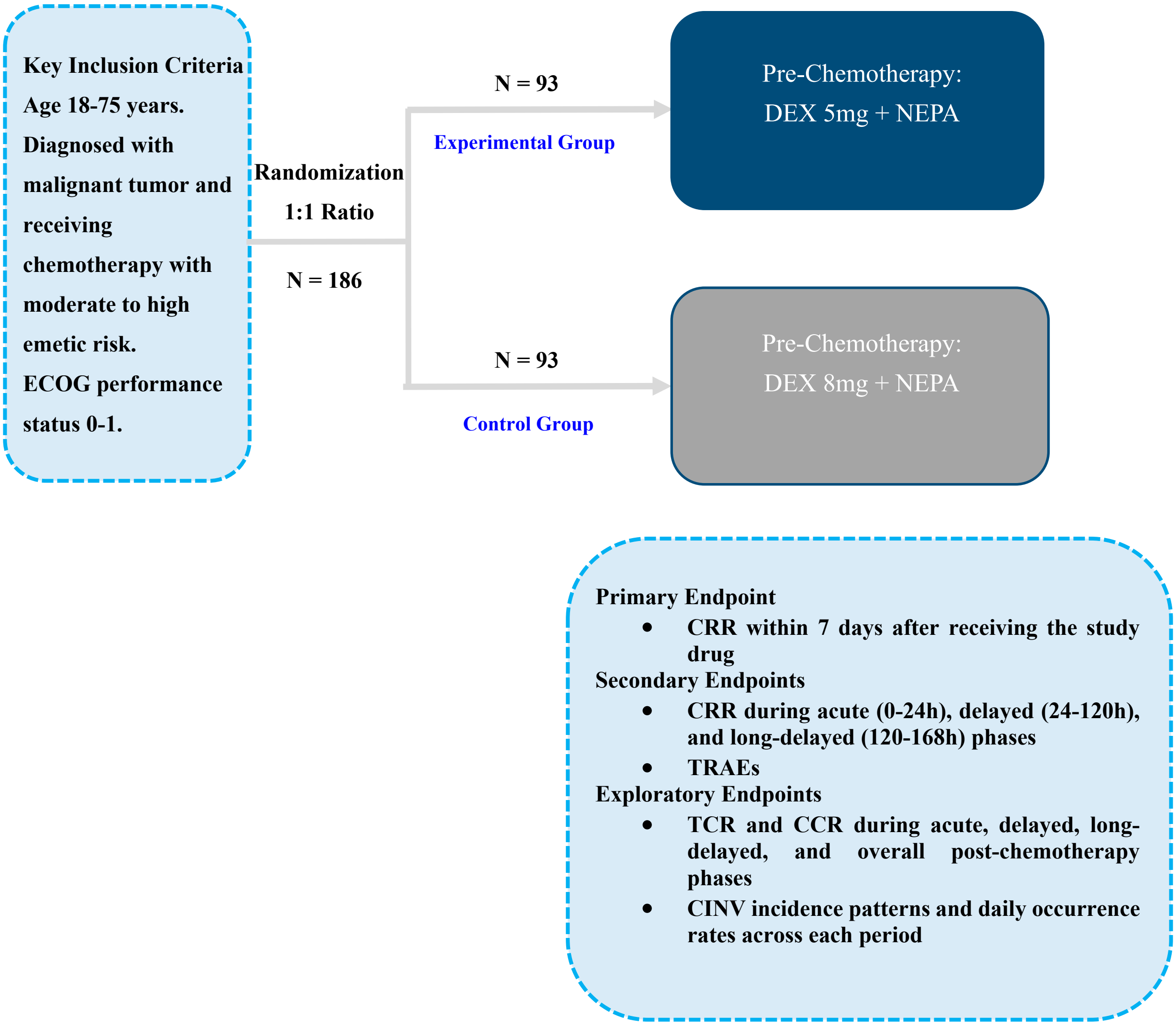

**Project**	**Screening Period**	**Baseline**	**Treatment Period**			
Visit Day	D-3~D0	D0	D1	D3	D5	D7
Informed Consent Signed	x					
Inclusion/Exclusion Criteria	x					
Randomization	x					
Demographic Data	x					
Medical History	x					
ECOG Performance Status	x					
Physical Examination	x	x	x	x	x	x
Vital Signsa[a]	x	x	x	x	x	x
Blood Glucose		x	x	x	x	x
Complete Blood Countb[b]	x				x	
Urinalysisc[c]	x					
Biochemical Profiled[d]	x	x			x	
Coagulation Functione[e]	x					
Pregnancy Testf[f]	x					
Thyroid Function Testsg[g]	x					
Viral Serologyh[h]	x					
Electrocardiogram (ECG)	x	x	x	x		
Echocardiography	x					
Doppler Ultrasound (Liver, Gallbladder, Pancreas, Spleen, Kidneys)	x					
PK Blood Sampling	x					
Drug Dispensation		x	x			
Past/Concomitant Medications	x					
Adverse Events		x	x	x	x	x
Compliance with Study Medicationi		x	x			
Rescue Therapy		x	x	x	x	x
Chemotherapy Assessment		x	x	x	x	x

Notes:

- *Safety follow-up will be conducted within 7 days after the last administration of netupitant/palonosetron capsules.
- a. Vital Signs: Including body temperature, blood pressure, pulse, and respiratory rate. Blood pressure should be measured as systolic and diastolic pressures after resting in a seated position for 5 minutes, and measurements should be taken at the same time and with the same sphygmomanometer whenever possible.
- b. Complete Blood Count: White blood cell count (WBC), neutrophil count (NEUT), neutrophil percentage, lymphocyte count (LYMPH), lymphocyte percentage, monocyte count, monocyte percentage, eosinophil count, eosinophil percentage, basophil count, basophil percentage, red blood cell count (RBC), hematocrit (HCT), mean corpuscular volume (MCV), mean corpuscular hemoglobin (MCH), mean corpuscular hemoglobin concentration (MCHC), hemoglobin (Hb), platelets (PLT).

- c. Urinalysis: Protein (PRO), glucose (GLU-U), microscopic WBC, microscopic RBC, casts, occult blood.
- d. Biochemical Profile: Aspartate aminotransferase (AST), alanine aminotransferase (ALT), gamma-glutamyl transferase (GGT), lactate dehydrogenase (LDH), total bilirubin (TBIL), direct bilirubin (DBIL), total protein (TP), albumin (ALB), alkaline phosphatase (ALP), triglycerides (TG), total cholesterol (TC), sodium (Na+), potassium (K+), chloride (Cl-), total calcium (Ca2+), glucose (GLU), urea (Urea) and/or blood urea nitrogen (BUN), creatinine (Cr), uric acid (UA).
- e. Coagulation Function: Prothrombin time (PT), activated partial thromboplastin time (APTT), thrombin time (TT), fibrinogen (FIB), international normalized ratio (INR), D-dimer (DD).
- f. Pregnancy Test: Only for women of childbearing potential. Blood pregnancy test will be conducted at the screening visit, and urine pregnancy test may be performed at other visits as deemed appropriate by the investigator.
- g. Thyroid Function: Free triiodothyronine (FT3), free thyroxine (FT4), thyroid-stimulating hormone (TSH).
- h. Viral Serology: Including hepatitis B surface antigen, hepatitis B surface antibody, hepatitis B e antigen, hepatitis B e antibody, hepatitis B core antibody, hepatitis C antibody, HIV antibody, syphilis antibody.
- i. Participants should ensure medication adherence as required by the protocol. If a dose is missed, the participant should continue taking the prescribed amount on the next day without doubling the dose.

1. Research Background and Rationale

1.1 Chemotherapy-Induced Nausea and Vomiting (CINV)

To this day, chemotherapy remains a cornerstone of cancer treatment. However, it is crucial to pay adequate attention to the associated adverse effects. The most common of these is chemotherapy-induced nausea and vomiting (CINV), which is directly related to a decline in quality of life and impairs patients' ability to manage daily activities[1, 2]. According to international guidelines (e.g., the American Society of Clinical Oncology (ASCO), the National Comprehensive Cancer Network (NCCN), the Multinational Association of Supportive Care in Cancer (MASCC), or the European Society for Medical Oncology (ESMO)), the current standard antiemetic treatment for patients receiving moderately emetogenic chemotherapy (MEC) to highly emetogenic chemotherapy (HEC) involves a three-drug regimen, typically including a neurokinin-1 (NK1) receptor antagonist (RA), a 5-hydroxytryptamine-3 (5-HT3) RA, and corticosteroids, with or without olanzapine. Among these, dexamethasone (DEX, oral/intravenous) is administered at preventive doses of 8 mg, 12 mg, or 20 mg on the first day (depending on the accompanying antiemetic drugs), followed by oral or intravenous administration of 8 mg for 2 to 3 days after chemotherapy [3-5].

Despite the long-standing use of DEX as part of the classic antiemetic regimen, its associated side effects should not be overlooked. These side effects may lead to metabolic abnormalities, glaucoma, cataracts, impaired wound healing, dyspepsia, hypertension, increased risk of infection, and increased risk of osteoporosis or osteonecrosis [6]. When combined with immunotherapy, the dosage of DEX also needs to be controlled. Netupitant and palonosetron capsules (NEPA) is a combination formulation consisting of netupitant and palonosetron, which can effectively target two key antiemetic pathways and may help reduce the dosage of DEX in CINV management. Therefore, we intend to explore the possibility of further reducing the total dosage of DEX when combined with NEPA before MEC/HEC administration.

1.2 Current Treatment and Research Status of CINV

The current guidelines recommend a multimodal regimen for preventing nausea and vomiting induced by moderately to highly emetogenic chemotherapy, typically including a 5-HT3 RA, an NK-1 RA, and multi-day dexamethasone, with or without olanzapine.

In a multicenter, placebo-controlled, Phase III study, Ito Y TT et al. conducted a non-inferiority trial comparing single-day versus multi-day standard-dose dexamethasone in patients receiving HEC. The results showed that when combined with an NK1-RA and palonosetron, dexamethasone could be tapered in the subsequent days without additional drug toxicities (46.9% vs. 44.0%, 95% CI, 212.6%-6.8%; P=0.007) [7]. However, this study reported that in the delayed phase, the complete control rate was higher in the 3-day dexamethasone group than in the single-day standard-dose group, suggesting that a 3-day dexamethasone regimen might be more appropriate for patients with risk factors for CINV. Subsequently, Celio L and Cortinovis D et al. validated the non-inferiority of shortening the dexamethasone course in patients receiving high-dose cisplatin, achieving study endpoints across all time intervals in this clinical trial [8]. As early as 2014, R.J. Gralla et al. conducted another Phase III study, demonstrating that NEPA, when combined with DEX, had a slight but consistent numerical advantage (2%-7%) in the total complete response (CR) rate over aprepitant plus palonosetron in consecutive cycles [9].

There are limited studies on reducing the dosage of DEX, and thus the appropriate dosage of antiemetics when NEPA is combined with MEC or HEC remains uncertain. A recent study published in 2023 showed that when patients received a single dose of 8 or 12 mg DEX on Day 1 before each HEC cycle, combined with single-day NEPA treatment, complete response rates of >95% were observed in both the acute and delayed phases, with all patients achieving a 100% CR rate from the third cycle onward [10]. A randomized Phase III study by Celio L et al. showed that neither of the two reduced-dose DEX groups demonstrated a significant impact of vomiting on the daily life of patients receiving high-dose cisplatin, with a DEX dose of 12 mg in DEX1 [11]. Another recently published Phase III study also confirmed the efficacy and safety of 12 mg DEX plus NEPA in elderly patients receiving cisplatin, with similar CR rates across treatment groups [12].

In summary, NEPA may help reduce the dosage of DEX in preventing CINV, optimize the use of DEX, and benefit patients with contraindications and side effects related to corticosteroids, offering a more convenient and durable alternative to the classic multimodal antiemetic regimen. Therefore, the combination of dexamethasone and NEPA for the treatment of CINV appears promising. This drug regimen has the distinct advantages of convenient administration and good compliance, and more evidence-based medical evidence is needed. Previously, we explored the efficacy and safety of single-day low-dose dexamethasone combined with single-day netupitant and palonosetron capsules for preventing CINV, with a total CR rate of approximately 85%. Therefore, our research team plans to further explore the efficacy and safety of single-day different low-dose dexamethasone combined with single or double doses of netupitant and palonosetron capsules in the treatment of CINV.

1.3 Potential Risks and Benefits

1.3.1 Known Potential Risks

Any drug or therapy at any stage of the study may present unforeseen and potentially severe toxicities and adverse effects. The primary risk for patients with chemotherapy-induced nausea and vomiting is the impact on food intake. For patients requiring radical concurrent chemotherapy for their tumors, CINV can lead to a decline in quality of life and physical function, resulting in reduced chemotherapy doses, delayed chemotherapy, or even termination of chemotherapy, thereby affecting the anti-tumor treatment process and efficacy, and potentially increasing mortality.

The current guidelines recommend a multimodal regimen for preventing nausea and vomiting induced by moderately to highly emetogenic chemotherapy, typically including a 5-HT3 RA, an NK-1 RA, and multi-day dexamethasone, with or without olanzapine. Although DEX has been used as part of the classic antiemetic regimen for many years, its associated side effects should not be overlooked. Vardy JL et al. conducted a survey on the side effects of dexamethasone for preventing CINV, summarizing moderate to severe adverse reactions including insomnia (45%), gastrointestinal symptoms (27%), anxiety and irritability (25%), increased appetite (18%), weight gain (17%), rash (15%), depression after discontinuation of treatment (7%), hiccups (7%), and oral candidiasis (3%). Among the 60 patients receiving MEC who participated in this survey, only 15 patients (25%, 95% CI 15-38%) reported no

side effects or only mild side effects, while 19 patients (32%, 95% CI 20-45%) reported at least three moderate to severe symptoms [13]. Given the high rate of reported symptoms, these side effects may outweigh the benefits of dexamethasone when used with moderately emetogenic chemotherapy. Another review also pointed out that corticosteroid therapy may lead to side effects such as metabolic abnormalities, central nervous system effects, glaucoma, cataracts, acne, impaired wound healing, dyspepsia, myopathy, hypertension, increased risk of infection, or hypothalamic-pituitary-adrenal axis suppression [14]. A dose-dependent sensation of perineal itching lasting several minutes after dexamethasone administration has also been reported in clinical trials [15].

Netupitant and palonosetron capsules (NEPA) is a combination formulation consisting of netupitant and palonosetron, which can effectively target two key antiemetic pathways and may help reduce the dosage of DEX in CINV management. A Phase III exploratory randomized controlled study by Zhang L and Lu S et al. in patients receiving HEC regimens showed the non-inferiority and similar safety of single-dose NEPA compared with the 3-day aprepitant plus dolasetron regimen (APR/GRAN group) when using the same dose of DEX. The most common adverse reactions were constipation (NEPA 8.0%, APR/GRAN 6.3%) and hiccups (NEPA 2.7%, APR/GRAN 1.4%), with fewer moderate adverse reactions reported in the NEPA group (APR/GRAN 10.8% versus NEPA 8.7%) [16]. Another Phase III exploratory randomized controlled study by Aapro M et al. showed that single-day NEPA combined with 12 mg DEX was more effective in preventing nausea and vomiting induced by MEC than single-day palonosetron combined with 20 mg DEX. The overall incidence, type, frequency, and intensity of adverse events were comparable between the two groups, with mild/moderate adverse events accounting for the majority of reported adverse events (85%), and the most common treatment-related adverse events were headache and constipation [17]. Multiple clinical trials and clinical observations have shown that NEPA can effectively prevent CINV with good safety. Given the complexity and high incidence of side effects associated with dexamethasone, we intend to explore the possibility of further reducing the total dosage of DEX when combined with NEPA before MEC/HEC administration.

Building on our previous exploration of the efficacy and safety of single-day low-dose dexamethasone combined with single-day netupitant and palonosetron capsules for preventing CINV, we have established an experimental group in this study. When combined with single-dose NEPA, we attempt to reduce the dose of

DEX from 8 mg to 5 mg, aiming to effectively prevent CINV while reducing the type, incidence, and intensity of potential adverse reactions. Although this approach may decrease the incidence and intensity of side effects associated with DEX, the reduced dosage of DEX may increase the risk of inadequate CINV control in the experimental group, potentially affecting chemotherapy compliance and quality of life.

A 2023 open-label, randomized controlled study by Gao A et al. in patients receiving HEC regimens showed the superiority and good safety of double-dose fosaprepitant over single-dose fosaprepitant in preventing delayed-phase CINV when using the same doses of palonosetron and dexamethasone [18]. Inspired by this, we conducted a second round of trials based on the previous treatment study, selecting patients from the control and experimental groups who did not achieve complete absence of nausea and vomiting in the first cycle for a second cycle of treatment. The primary and secondary endpoints in the second cycle were self-controlled with those in the first cycle, exploring the efficacy and safety of single-day different low-dose dexamethasone combined with double-dose standard NEPA in preventing nausea and vomiting induced by moderately to highly emetogenic chemotherapy. Although NEPA has shown good safety in multiple clinical trials and clinical observations, there are limited studies on the use of multiple doses of netupitant and palonosetron capsules in preventing nausea and vomiting induced by MEC/HEC, and no studies have been conducted on the use of NEPA after chemotherapy. The instructions also state that NEPA should not be used to prevent nausea and vomiting after chemotherapy, making this an off-label use with unknown risks and the potential for unforeseen and severe toxicities and adverse effects.

Based on the existing safety clinical data of dexamethasone and netupitant and palonosetron capsules, adverse events reported by similar products, and the characteristics of the study population, the following measures have been taken in risk control: (1) Strictly adhering to the inclusion and exclusion criteria for participants, excluding those with severe, uncontrolled comorbidities, as well as diseases or clinical conditions that may interfere with the evaluation of the drugs; (2) Closely monitoring adverse events (AEs) in participants during the study, including AEs and AEs of special concern, such as significant gastrointestinal reactions and new drug-induced rashes, and providing suggested management measures; (3) Permitting rescue treatment in the trial at the investigator's discretion, including reducing the dose of dexamethasone, substituting other antiemetic drugs, delaying chemotherapy, reducing chemotherapy doses, or temporarily discontinuing chemotherapy.

1.3.2 Known Potential Benefits

The potential benefits for participants in this study are the reduction of chemotherapy-induced nausea and vomiting in a more convenient and durable manner, thereby further improving quality of life and avoiding the need to reduce chemotherapy doses or delay chemotherapy, or even suspend chemotherapy. Indirect benefits include reducing adverse reactions associated with antiemetic drugs, such as insomnia, gastrointestinal symptoms, anxiety and irritability, rash, increased risk of infection, etc. Completing chemotherapy at full doses, full courses, and on schedule will also improve treatment outcomes and prognosis for cancer patients.

2. Research Objectives and Endpoint Goals

2.1 Research Objectives

2.1.1 Primary Objective

To explore the efficacy of single-day different low-dose dexamethasone combined with single dose of standard NEPA in preventing nausea and vomiting induced by moderately to highly emetogenic chemotherapy.

2.1.2 Secondary Objectives

To evaluate the safety of single-day different low-dose dexamethasone combined with single dose of standard NEPA in preventing nausea and vomiting induced by moderately to highly emetogenic chemotherapy.

2.2 Research Indicators

2.2.1 Primary Endpoint

Proportion of subjects with complete response (defined as no vomiting and no rescue medication within 7 days after receiving the study drug).

2.2.2 Secondary efficacy Endpoints:

CRR during acute (0-24h), delayed (24-120h), and long-delayed (120-168h) phases

2.2.3 Secondary Safety Endpoints

Treatment related adverse events (evaluated using the National Cancer Institute Common Terminology Criteria for Adverse Events (NCI CTCAE) version 5.0).

2.2.4 Exploratory endpoints

1. TCR and CCR during acute, delayed, long-delayed, and overall post-chemotherapy phases.
2. CINV incidence patterns and daily occurrence rates across each period.

3. Study Design

This study is a randomized controlled non-inferiority clinical trial conducted at Xiamen University Zhongshan Hospital, which will be registered at Chinese Clinical Trial Registry (ChiCTR) prior to participant enrollment. The study groups include the control group and the experimental group. The trial consists of screening period and treatment period.

3.1 Screening Period

Inclusion and exclusion criteria are evaluated. Those who meet the criteria can enter the treatment period.

3.2 Treatment Period

(1) Control Group: 8 mg of dexamethasone is administered intravenously 0.5 hours before chemotherapy, and 1 tablet of netupitant and palonosetron hydrochloride capsules (each containing 0.3 g of netupitant and 0.5 mg of palonosetron hydrochloride) is taken orally 1 hour before chemotherapy.

(2) Experimental Group: 5 mg of dexamethasone is administered intravenously 0.5 hours before chemotherapy, and 1 tablet of netupitant and palonosetron hydrochloride capsules (each containing 0.3 g of netupitant and 0.5 mg of palonosetron hydrochloride) is taken orally 1 hour before chemotherapy.

Stratification Factors: Gender, age, patient's risk factors for CINV, ECOG score, tumor type, distant metastasis of the tumor, emetogenic risk of the chemotherapy regimen, smoking history, and drinking history, etc.

Drug Discontinuation Criteria During Treatment: Subjects should discontinue the drug if they experience severe adverse events or specific types of adverse events, such as severe drug intolerance or allergic reactions.

4. Selection and Withdrawal of Study Subjects

4.1 Inclusion Criteria

Subjects must meet all the following inclusion criteria to be enrolled in this study:

1. Age 18-75 years old.
2. Diagnosed with malignant tumors and undergoing chemotherapy regimens with moderate to high emetogenic risk.
3. ECOG score 0-1.
4. Expected survival of ≥ 12 weeks at screening and able to receive the current anti-tumor treatment for at least one cycle.
5. Adequate organ function meeting the following criteria:

   a: Neutrophil count ≥ 1.5 × 109/L;

   b: Platelet count ≥ 100×109/L;

   c: In patients without known liver metastases, aspartate aminotransferase ≤ 3 × ULN and/or alanine aminotransferase ≤ 3 × ULN (for patients with liver metastases, it can be relaxed to ≤ 5 × ULN);
6. Eligible subjects of childbearing age must agree to use reliable contraceptive measures throughout the study period (including male or female condoms, contraceptive foams, gels, films, creams, suppositories, abstinence, and intrauterine devices, etc.), except for female subjects who have undergone

hysterectomy, bilateral salpingectomy, bilateral tubal ligation, or have been postmenopausal for more than one year, and male subjects who have undergone bilateral vasectomy or ligation.

7. Voluntary participation in this study, signing the informed consent form, and good compliance.

4.2 Exclusion Criteria

Subjects with any of the following conditions are not eligible for this study:

1. Received radiotherapy within one week before chemotherapy.
2. Suffer from other diseases that cause nausea or vomiting except those caused by tumor chemotherapy drugs, including but not limited to gastrointestinal obstruction and central nervous system malignancies.
3. Experienced vomiting, nausea, or mild nausea within 24 hours before treatment.
4. Concurrent use of corticosteroids or any other possible antiemetic drugs.
5. Contraindications to corticosteroids.
6. Cognitive impairment (such as dementia or severe learning difficulties) that prevents completion of nausea and vomiting-related evaluation scales, etc.
7. Use of antipsychotic drugs within 30 days before the start of treatment or during treatment.
8. Severe and uncontrolled diseases affecting the liver, kidneys, cardiovascular, respiratory, endocrine systems, or the central nervous system.
9. Allergy to the study drugs.
10. Pregnant or lactating women.
11. Participated in any other clinical study of investigational drugs or devices within three months before screening.
12. The investigator deems that participation in the trial poses a significant risk to the subject's health or safety or may affect the assessment of efficacy for other reasons.

4.3 Withdrawal Criteria

Patients will be withdrawn from the study if any of the following occurs:

1. The subject withdraws informed consent to participate in the study or requests to withdraw from the study and refuses further follow-up.
2. The investigator deems it necessary to withdraw the subject from the study for other reasons, such as the subject losing the ability to express their will due to imprisonment or isolation, etc.
3. Loss to follow-up.
4. Serious adverse events where risks outweighed benefits.
5. The investigator or regulatory authority requests termination of the study.

5. Study Medications

5.1 Basic Information of Medications

- **Dexamethasone Sodium Phosphate Injection**
  - Manufacturer: Chendxin Pharmaceutical Co., Ltd.
  - Dosage form: Injection
  - Route of administration: Intravenous injection
  - Specification: 1 ml: 5 mg
  - Storage and stability: Protect from light, keep tightly closed, store at or below 25°C (do not freeze), shelf life of 24 months.
- **Netupitant and Palonosetron Hydrochloride Capsules**
  - Trade name: Akynzeo
  - Manufacturer: Helsinn Birex Pharmaceuticals Ltd.
  - Dosage form: Hard capsules
  - Route of administration: Oral

- Specification: Each hard capsule contains 0.3 g of netupitant (0.1 g per tablet × 3 tablets) and 0.5 mg of palonosetron hydrochloride (calculated as C19H24H2O, 1 soft capsule).
- Storage and stability: Store at room temperature (below 30°C), sealed, shelf life of 36 months.

5.2 Dosing Regimen

5.2.1 Drug Dose

- **Control Group**: 8 mg of dexamethasone is given intravenously 0.5 hours before chemotherapy, and 1 capsule of netupitant and palonosetron hydrochloride capsules (each capsule contains 0.3 g of netupitant and 0.5 mg of palonosetron hydrochloride) is taken orally 1 hour before chemotherapy.
- **Experimental Group**: 5 mg of dexamethasone is given intravenously 0.5 hours before chemotherapy, and 1 capsule of netupitant and palonosetron hydrochloride capsules (each capsule contains 0.3 g of netupitant and 0.5 mg of palonosetron hydrochloride) is taken orally 1 hour before chemotherapy.

5.2.2 Drug Discontinuation Criteria During Treatment

Drug discontinuation is recommended when patients experience severe adverse events or specific types of adverse events, such as severe drug intolerance or allergic reactions.

5.3 Concomitant Medication

Concomitant medication/treatment is given at the discretion of the investigator based on the interests of the subjects. All treatments and medications (excluding solvent medications) used within 28 days before signing the informed consent form until the end of the safety follow-up period should be strictly recorded in the eCRF in accordance with GCP regulations. If the subjects experience adverse events (AE), they should be closely observed and actively treated symptomatically if necessary. The medical documents should be fully recorded and the drugs and non-drug treatments used should

be recorded and explained in the eCRF. After the above collection period, only concomitant medications and non-drug treatments related to the study drug for AE/SAE should be recorded.

5.3.1 Permitted Medications Prior to Study

Participants who have received corticosteroids or any other antiemetic medications before screening but have discontinued them for at least 7 days at the time of screening.

5.3.2 Permitted Medications During the Study

Metoclopramide is uniformly used as the first-choice rescue medication during the study. Refractory CINV is managed in accordance with guidelines and the attending physician's opinion.

5.3.3 Prohibited or Cautiously Used Medications and Treatments During the Study

During the study, the use of any other antiemetic medications other than metoclopramide (unless deemed necessary by the investigator) is prohibited.

5.4 Medication Adherence

Medication adherence in this study is the responsibility of the principal investigator. Patients who do not meet the criteria for analysis will not be included in the final analysis.

6 Study Procedures

This study is divided into three phases: screening phase, treatment phase, and follow-up phase.

6.1 Screening Phase

The screening phase begins with the signing of the informed consent form (ICF) by participants who meet the criteria of this study and ends with enrollment or screening failure. Participants who discontinue the study after signing the informed consent form but before enrollment will be considered as "screening failures."

The following screening steps must be completed within 14 days before starting the study drug treatment:

Obtain written informed consent signed by the participant;

Collect demographic information;

Collect medical and treatment history;

Collect medication history;

Conduct physical examination and ECOG PS score;

Laboratory tests, such as screening for infectious diseases, routine urinalysis, and stool examination;

Collect relevant imaging results for tumor baseline assessment, such as chest and abdominal CT, brain MRI, bone ECT, etc.;

Evaluate concomitant medications and treatments currently in use.

The following screening steps must be completed on the day of starting the study drug treatment:

Physical examination, vital signs, and ECOG PS score;

Laboratory tests, such as complete blood count, biochemical profile, coagulation function, etc.

6.2 Treatment Phase

The treatment phase begins with the administration of the study drug and continues until the completion of this round of cancer treatment, disease progression, death, loss to follow-up, or initiation of new anti-cancer treatment, whichever occurs first. Participants should start receiving the study drug within 24 hours of enrollment.

6.3 Follow-up Phase

6.3.1 End of Study Treatment/Withdrawal Visit

Participants should complete the end of study treatment/withdrawal visit when they discontinue the study. If participants do not have relevant test results within 7 days before the end of treatment, the tests specified in the protocol should be conducted within 7 days after the end of treatment. The following items should be completed at the end of treatment/withdrawal: [Complete Blood Count] [Biochemical Profile] [Vital Signs] [Physical Examination] [ECOG Score] [Adverse Events] [Concomitant Medications/Treatments].

6.3.2 Safety Follow-up Visit

Seven days (±3 days) after the last administration of the study drug, regardless of whether the participant has started new anti-cancer treatment, the participant should return to the research center for a safety follow-up visit and complete the following safety assessment indicators. If a participant is unable to resume study drug administration due to an AE, the last administration date is determined as the last dose date. If this date is more than 7 days (+3 days) from the time they were judged to need to discontinue study treatment, this situation does not need to be recorded as a protocol deviation, but it is recommended that the participant return to the research center for a safety follow-up visit as soon as possible. The following items should be completed at the 7-day visit after the last administration of the study drug: [Complete Blood Count] [Biochemical Profile] [Vital Signs] [Physical Examination] [ECOG Score] [Adverse Events] [Concomitant Medications/Treatments].

7 Efficacy Evaluation

7.1 Primary Efficacy Endpoint

Complete response rate (no vomiting and no use of rescue medication within 7

days after administration of the study drug).

7.2 Secondary Efficacy Endpoints

CRR during acute (0-24h), delayed (24-120h), and long-delayed (120-168h) phases.

7.3 Exploratory Efficacy Endpoints

TCR and CCR during acute, delayed, long-delayed, and overall post-chemotherapy phases.

CINV incidence patterns and daily occurrence rates across each period.

8 Safety Evaluation

Safety evaluation includes the recording of adverse events (AEs) and serious adverse events (SAEs) from the time participants sign the informed consent form until the end of the study, through vital signs, physical examination, laboratory tests, electrocardiogram, echocardiogram, abdominal ultrasound, etc. The safety of the study drug is evaluated according to the Common Terminology Criteria for Adverse Events (CTCAE) version 5.0, and the incidence and severity of nausea and vomiting events are recorded in accordance with the NCCN (2024.V1) Clinical Practice Guidelines. Participants should be closely monitored for changes in symptoms and signs after drug administration during the study. Any AEs that occur should be promptly managed to ensure the safety and well-being of the participants. After timely management of AEs, their diagnosis (or symptoms if no diagnosis is made), onset and resolution dates, severity, relationship to the study drug, whether treatment was required, management measures, and outcomes should be recorded, followed by analysis, evaluation, and statistical analysis of the AEs.

All participants must undergo comprehensive physical and laboratory examinations during the study, including measurement of temperature, pulse, respiration, blood pressure, complete blood count, urine routine, biochemical profile,

coagulation function, 12-lead electrocardiogram, echocardiogram, and abdominal ultrasound. If clinically significant abnormal changes occur, they should be closely monitored and managed until they return to normal or baseline levels or stabilize.

8.1 Physical Examination and Vital Signs

Physical examination is conducted by the study physician and includes the following: general condition, skin and mucous membranes, lymph nodes, head, eyes, ears, nose, throat, neck, chest, abdomen, musculoskeletal system, neurological reflexes, respiratory system, cardiovascular system, genitourinary system, and mental status. Weight should be measured each time a physical examination is conducted, while height needs to be measured only once during the screening period.

Vital signs include the following: temperature, blood pressure, respiratory rate, and heart rate.

The ECOG score is assessed by the study physician according to the "Performance Status Score (ECOG)" in Appendix I.

8.2 Laboratory Tests

Laboratory test samples will be collected at the time points specified in the "Clinical Trial Flowchart."

Complete blood count: A complete blood count is repeated twice after each chemotherapy cycle, and the frequency of testing can be increased if there are abnormal laboratory results.

Biochemistry: Biochemical tests are repeated once a week, and the frequency of testing can be increased if there are abnormal laboratory results.

8.3 Nausea and Vomiting Assessment

Investigators will record the incidence and severity of nausea and vomiting events according to the NCCN (2024.V1) and CTCAE (V5.0) Clinical Practice Guidelines.

8.4 Adverse Events (AEs)

8.4.1 Definition of Adverse Events

An adverse event is any untoward medical occurrence in a participant in a clinical trial after signing the informed consent form, but it does not necessarily have a causal relationship with the study drug. An adverse event can be any unexpected, unfavorable symptom, sign, disease, or abnormal laboratory finding, regardless of whether it is related to the study drug. Adverse events include the following situations: 1) pre-existing medical conditions/diseases before the start of study treatment, which are only considered adverse events if they worsen after the initiation of the study drug; 2) any new adverse events; 3) abnormal laboratory values or findings, which are considered adverse events only if they are deemed clinically significant.

8.4.2 Criteria for Assessing the Severity of Adverse Events

Refer to the grading criteria for drug adverse events in CTCAE version 5.0. If an adverse event not listed in the CTCAE version 5.0 tables occurs, the following criteria may be used:

- Grade I: Mild; no clinical symptoms or only slight clinical symptoms; detected only clinically or diagnostically; no treatment required.
- Grade II: Moderate; requiring minimal, topical, or non-invasive treatment; limitation of instrumental activities of daily living (ADL), which include cooking, shopping, making phone calls, managing finances, etc.
- Grade III: Severe or medically important but not immediately life-threatening; resulting in hospitalization or prolonged hospital stay; causing disability; limitation of self-care ADL, which include bathing, dressing, undressing, eating, toileting, taking medications, etc., but not bedridden.
- Grade IV: Life-threatening consequences; requiring urgent treatment.
- Grade V: Death related to the adverse event.

8.4.3 Criteria for Assessing the Relationship Between Adverse Events and the Study Drug

All adverse events, including any unexpected clinical manifestations that occur after signing the informed consent form, regardless of whether they are related to the study drug or even whether the drug was administered, should be reported as adverse events. All adverse events must be reported in the form of a clinical report. Any discomfort reported by the patient during the treatment period or any abnormal changes in objective laboratory test indicators should be accurately recorded, along with the severity, duration, management measures, and outcomes of the adverse events. Investigators should also comprehensively assess the relationship between adverse events and the study drug, using a five-category classification method to evaluate the potential association between adverse events and the study medication: "definitely related," "probably related," "probably not related," "definitely not related," and "unable to determine." "Definitely related," "probably related," and "unable to determine" are all classified as drug adverse reactions. When calculating the incidence of adverse events, the sum of these two categories is used as the numerator, and the total number of participants used for safety evaluation is used as the denominator. The criteria for judgment are as follows:

Table 2: Criteria for Assessing the Relationship Between AEs and the Investigational Drug

Category	Criteria
Definitely Related	The event occurs within a plausible time frame after drug administration, aligns with the known reaction profile of the drug, improves upon discontinuation, and recurs upon re-administration.
Possibly Related	The event occurs within a plausible time frame after drug administration but does not align with the known reaction profile of the drug. The subject's clinical condition or other treatments may also contribute.
Possibly Unrelated	The event does not occur within a plausible time frame after drug administration and does not align with the known reaction profile of the drug. The subject's clinical condition or other treatments may contribute.
Definitely Unrelated	The event does not occur within a plausible time frame after drug administration and does not align with the known reaction profile of the drug. The subject's clinical condition or other treatments are likely the cause, and the event resolves with disease improvement or discontinuation of other treatments.
Unable to Determine	The event's timing relative to drug administration is unclear, and it resembles the known reaction profile of the drug or could be caused by concomitant medications.

8.4.4 Recording and Reporting of Adverse Events

Researchers should meticulously document any adverse events that occur in the subjects, including: a description of the adverse event and all related symptoms, the time of occurrence, severity, end time, measures taken, and the final outcome.

From the time the subject signs the informed consent form until 28 days after the last dose, drug safety assessments are required. All adverse events (both serious and non-serious) should be recorded on the adverse event reporting page of the case report form, using precise medical terminology. Follow-up should continue until the adverse event returns to Grade I or below, or until the investigator deems further follow-up unnecessary (e.g., clinical stability is achieved).

8.5 Serious Adverse Events (SAEs)

8.5.1 Definition of Serious Adverse Events

A serious adverse event (SAE) is any medical event occurring during a clinical trial that requires hospitalization, prolongs hospital stay, results in disability, affects work capacity, is life-threatening, or leads to death, or causes congenital anomalies. Adverse events meeting one or more of the following criteria are considered SAEs:

- Resulting in death;
- Life-threatening (defined as an immediate risk of death at the time of the event);
- Requiring hospitalization or prolonging hospital stay;
- Causing permanent or significant disability/functional impairment;
- Resulting in congenital anomalies or birth defects;
- Important medical events: These adverse events may not be life-threatening, but based on medical judgment, they may endanger the subject and require medical or surgical intervention to prevent any of the above outcomes.

Investigators should promptly report all SAEs, including clinical diagnosis, treatment, and outcomes, and follow up until the event is resolved, alleviated, or stabilized. Detailed records should be maintained in the original medical records and eCRF forms (including the adverse event report form; if death occurs, a death report form should be completed), and an SAE report form should be filed.

8.5.2 Tumor Disease Progression

Tumor disease progression is defined as the deterioration of the subject's condition due to tumor disease progression, including radiological progression and the progression of clinical symptoms and signs. The appearance of new metastatic sites of the primary tumor or the progression of existing metastatic sites is considered disease progression. Events that are life-threatening, require hospitalization or prolong hospital

stay, or result in permanent or significant disability/functional impairment, congenital anomalies, or birth defects due to symptoms and signs of disease progression are not reported as SAEs. If there is any uncertainty about whether an SAE is due to disease progression, it should be reported as an SAE.

8.6 Research-Related Injury and Compensation

While this study will be conducted in strict compliance with applicable laws and regulations, adverse reactions may still occur. In the event of research-related injury, the investigator will provide immediate medical care and treatment. Compensation for damages will be provided in accordance with legal requirements if negligence is determined. The investigator will make every effort to prevent and manage any potential harm resulting from study participation.

9 Management of the Research Project

9.1 Ethical Standards and Informed Consent

9.1.1 Ethical Standards

This clinical trial must adhere to the Declaration of Helsinki, the "Good Clinical Practice" (GCP) guidelines issued by the NMPA, and relevant regulations. Before the start of the trial, approval from the hospital's ethics committee must be obtained. Any modifications to the trial protocol during the clinical research must be reported to the ethics committee for record-keeping. Investigators are responsible for submitting interim reports to the ethics committee in accordance with their requirements and informing them of the trial's conclusion.

9.1.2 Informed Consent

Subjects must provide informed consent before participating in this observational study. Participants may optionally consent to future use of de-identified data for CINV-

related research, with separate approval required for new studies.

9.2 Drug Management

The drugs used in this study are already marketed in clinical practice, and no other medications are provided.

9.3 Protocol Amendments

No one other than the sponsor is permitted to amend the protocol. Any necessary changes must be made in the form of protocol revisions and submitted to the ethics committee for approval or record-keeping after obtaining signatures from the sponsor and the principal investigator. All modifications must be detailed in the protocol.

9.4 Monitoring

The sponsor appoints personnel with appropriate medical, pharmaceutical, or related academic qualifications, who have undergone necessary training and are familiar with GCP and relevant regulations, to oversee the collection of information for this clinical trial. They ensure that trial records and reports are accurate, complete, and error-free, and that the trial adheres to the approved protocol, GCP, and relevant regulations. Monitoring of adverse events and serious adverse events in clinical trials ensures that all such events are accurately, reliably, and promptly recorded and reported.

9.5 Quality Control and Assurance

Clinical research units must be designated by the NMPA as qualified for clinical research.

9.6 Data Management

9.6.1 Data Collection

This study employs eCRF for the collection of research data.

9.6.2 Data Management and Quality Control

All participant data will be de-identified using unique codes, stored in password-protected systems, and accessible only to authorized study personnel. Identifiable information will not be shared externally without separate participant consent, except as required by regulatory authorities. Data will be retained for 5 years post-trial completion before secure destruction. To ensure the authenticity and reliability of clinical trial data and enhance data quality, clinical monitors will follow standard operating procedures to review the completeness, consistency, and accuracy of trial data in the clinical database during the project. They will guide research institution staff in supplementing or correcting problematic data as necessary. At the end of the trial, data managers and medical personnel will conduct a final quality control review of all database data, summarizing all protocol deviations and violations that occurred during the trial. Once the data in the database meet quality requirements, the database will be locked, and data managers will export the data for statistical analysis by the statistics department.

9.6.3 Data Review

Investigators must retain the original files of each patient participating in the trial, including study medical records and visit records (inpatient or outpatient medical records), which include demographic and medical information, laboratory data, electrocardiograms, and results of any other tests or evaluations. All information on the eCRF must originate from the original files in the patient's records. Investigators must also retain the informed consent forms signed by the patients.

9.7 Protocol Deviations

All requirements specified in the study protocol must be strictly followed. Any intentional or unintentional deviations from or violations of the trial protocol and GCP principles are classified as protocol deviations or violations.

9.8 Publication of Study Results

All articles and reports related to the trial must be approved by the investigators before publication.

10 Data Analysis and Statistical Methods

10.1 Sample Size Estimation

Based on previous studies of 8-12 mg DEX plus NEPA for CINV prevention [8,10], a CRR of 80% was assumed for the control group (8D DEX group). The non-inferiority margin was set at 15%. For the sample size calculation, the CRR in the experimental group (5D DEX group) was also assumed to be 80% under the null hypothesis of non-inferiority. Using a normal approximation method for non-inferiority testing of proportions (Z-test, unpooled, one-sided α=0.05, power=80%), a total of 176 participants (88 per group) was calculated to be sufficient to demonstrate non-inferiority. To account for a potential 5% attrition rate, the enrollment was increased to 186 participants (93 per group). The sample size calculation was performed using PASS 2021 software.

10.2 Randomization and masking

Computer-generated randomization (SPSS v26.0) assigned 186 patients using a reproducible seed number (2000000). A sequence of random values ranging from 0 to 1 was generated for all 186 participants. Allocation was performed strictly in the order of this pre-generated sequence: the first 93 participants corresponding to the smallest

random values were assigned to the 5D DEX group, and the remaining 93 to the 8D DEX group. This sequential assignment based on a computer-generated random list ensured the randomness of allocation. Investigators enrolled eligible participants and allocated them according to this computer-generated randomization sequence. This open-label study maintained full allocation transparency to all investigators and participants throughout the single-cycle observation.

10.3 Analysis Populations

The analysis populations in this study include the modified Intention-To-Treat (mITT) and the Per-Protocol Set (PPS).

- **Modified Intention-To-Treat (mITT)**: All subjects who are enrolled and receive at least one treatment will be included in this analysis set. mITT is the primary analysis set for efficacy analysis in this study.
- **Per-Protocol Set (PPS)**: Includes only participants who fully adhered to the trial protocol (e.g., completed treatment, complied with visits, and had no major violations).

10.4 Handling of Missing Data

If the baseline date is missing, it will be imputed with the last available date. For missing dates after baseline due to early withdrawal, right censoring will be applied. The censoring date will be determined by the principal investigator after a case-by-case review of post-treatment laboratory tests.

Other missing baseline indicators and safety data will not be imputed.

10.5 General Principles for Efficacy Analysis

In this study, unless otherwise specified, efficacy data will be descriptively summarized according to the following general principles:

- Demographic and clinical characteristics were analyzed using descriptive statistics, with categorical variables expressed as frequencies (percentages) and continuous variables as median (interquartile range).

- The efficacy endpoints (CRR, TCR, and CCR) were analyzed for noninferiority using a stratified Mantel-Haenszel approach to compare dose cohorts, adjusting for age. The combined risk difference and its two-sided 95% confidence interval were calculated. Noninferiority was concluded if the lower confidence limit exceeded the pre-specified margin of -15%. Changes in response within subjects across different treatment phases were assessed using McNemar's test. P values for noninferiority tests were reported as one sided, whereas other P values were two sided.
- Group comparisons employed chi-square/Fisher's exact tests for binary variables, Mann-Whitney U test for continuous variables, and Kruskal-Wallis test for multicategorical data.

10.6 General Principles for Safety Analysis

Safety analysis includes:

- Analysis of patient discontinuation, dose reduction, or suspension due to adverse events;
- Incidence and severity of adverse events;
- Analysis of the relationship between adverse events and the drug;
- Analysis of the outcomes of adverse events;
- Analysis of serious adverse events;
- Descriptive statistical summary of laboratory, vital signs, and electrocardiogram data;
- Incidence of abnormal laboratory indicators;
- Analysis of positive abnormal changes in laboratory indicators, vital signs, and other data compared to baseline.

11 Dropout of Subjects

All subjects who have signed the informed consent form and are screened as eligible to enter the trial have the right to withdraw from the study at any time. Subjects

who withdraw for any reason before completing the clinical trial and cannot be evaluated for safety and efficacy are considered dropouts (subjects who withdraw due to disease progression with clear medical evidence after enrollment are not considered dropouts). When a subject drops out, the investigator must record the reason for dropout in the CRF, complete all possible assessment items, and carefully document the last visit record in the CRF. For subjects who drop out due to adverse reactions and are finally determined to be related to the study drug after follow-up, this should be recorded in the CRF and notified to the investigator. Subjects who only underwent screening but did not receive a drug number and withdrew from the study are not considered dropouts. All enrolled subjects with at least one medication record will be included in the safety set and should be analyzed in the safety evaluation.

Subjects who withdraw from the study cannot re-enter the study, and their numbers cannot be reused.

12 Study Timeline

- Enrollment of the first subject: October 2024
- Estimated enrollment of the last subject: August 2025
- Estimated study completion date: August 2025

References

1. Sharma R, Tobin P, Clarke SJ. Management of chemotherapy-induced nausea, vomiting, oral mucositis, and diarrhoea. *Lancet Oncol.* 2005;6(2):93-102. doi:10.1016/S1470-2045(05)01735-3.

2. Haiderali A, Menditto L, Good M, Teitelbaum A, Wegner J. Impact on daily functioning and indirect/direct costs associated with chemotherapy-induced nausea and vomiting (CINV) in a US population. *Support Care Cancer*. 2010;19(6):843-851. doi:10.1007/s00520-010-0915-9.

3. Roila F, Molassiotis A, Herrstedt J, Aapro M, Gralla RJ, Bruera E, et al. 2016 MASCC and ESMO guideline update for the prevention of chemotherapy- and radiotherapy-induced nausea and vomiting and of nausea and vomiting in advanced cancer patients. *Ann Oncol*. 2016;27:v119-v133. doi:10.1093/annonc/mdw270.

4. Hesketh PJ, Kris MG, et al. Antiemetics: American Society of Clinical Oncology Clinical Practice Guideline Update. *J Clin Oncol.* 2017;35:3240-3261. doi:10.1200/JCO.2017.74.4789.

5. NCCN Clinical Practice Guidelines in Oncology: Antiemesis (2024 Version 1). NCCN Guidelines. Available at: http://www.nccn.org.

6. Grunberg SM. Antiemetic activity of corticosteroids in patients receiving cancer chemotherapy: dosing, efficacy, and tolerability analysis. *Ann Oncol*. 2007;18:170-176. doi:10.1093/annonc/mdl347.

7. Ito Y, Minatogawa H, et al. Placebo-Controlled, Double-Blinded Phase III Study Comparing Dexamethasone on Day 1 With Dexamethasone on Days 1 to 3 With Combined Neurokinin-1 Receptor Antagonist and Palonosetron in High-Emetogenic Chemotherapy. *J Clin Oncol*. 2018;36:1000-1006. doi:10.1200/JCO.2017.74.4375.

8. Celio L, Cortinovis D, Cogoni AA, Cavanna L, Martelli O, Carnio S, et al. Dexamethasone-Sparing Regimens with Oral Netupitant and Palonosetron for the Prevention of Emesis Caused by High-Dose Cisplatin: A Randomized Noninferiority Study. *Oncologist*. 2021;26(10):e1854-e1861. doi:10.1002/onco.13851.

9. Gralla RJ, Bosnjak SM, Hontsa A, Balser C, Rizzi G, Rossi G, et al. A phase III study evaluating the safety and efficacy of NEPA, a fixed-dose combination of netupitant and palonosetron, for prevention of chemotherapy-induced nausea and vomiting over repeated cycles of chemotherapy. *Ann Oncol*. 2014;25(7):1333-1339. doi:10.1093/annonc/mdu096.

10. Agre SA, et al. Retrospective Evaluation of a Dexamethasone Sparing Antiemetic Regimen: An Antiemetic Prophylaxis Study on NEPA (Netupitant Plus Palonosetron) for Preventing Chemotherapy-Induced Nausea and Vomiting (CINV) in Cancer Patients. *Cureus*. 2021;13(12):e19866. doi:10.7759/cureus.49763.

11. Celio L, Cortinovis D, Cogoni AA, Cavanna L, Martelli O, Carnio S, et al. Evaluating the impact of chemotherapy-induced nausea and vomiting on daily functioning in patients receiving dexamethasone-sparing antiemetic regimens with NEPA (netupitant/palonosetron) in the cisplatin setting: results from a randomized phase 3 study. *BMC Cancer*. 2022;22(1):10018. doi:10.1186/s12885-022-10018-3.

12. Celio L, Bartsch R, Aapro M. Dexamethasone-sparing regimens with NEPA (netupitant/palonosetron) for the prevention of chemotherapy-induced nausea and vomiting in older patients (>65 years) fit for cisplatin: A sub-analysis from a phase 3 study. *J Geriatr Oncol*. 2023;14(6):101537. doi:10.1016/j.jgo.2023.101537.

13. Vardy J, Chiew KS, Galica J, Pond GR, Tannock IF. Side effects associated with the use of dexamethasone for prophylaxis of delayed emesis after moderately emetogenic chemotherapy. *Br J Cancer*. 2006;94(7):1011-1015. doi:10.1038/sj.bjc.6603048.

14. Rutgeerts PJ. Review article: the limitations of corticosteroid therapy in Crohn's disease. *Aliment Pharmacol Ther*. 2001;15(10):1515-1525. doi:10.1046/j.1365-2036.2001.01060.x.

15. Zaglama NE, et al. Single, High-Dose Intravenous Dexamethasone as an Antiemetic in Cancer Chemotherapy. *Oncology*. 1986;43(1):27-32. doi:10.1159/000226099.

16. Zhang L, Lu S, et al. A randomized phase III study evaluating the efficacy of single-dose NEPA, a fixed antiemetic combination of netupitant and palonosetron, versus an

aprepitant regimen for prevention of chemotherapy-induced nausea and vomiting (CINV) in patients receiving highly emetogenic chemotherapy (HEC). *Ann Oncol*. 2018;29(2):452-458. doi:10.1093/annonc/mdx698.

17. Aapro M, et al. A randomized phase III study evaluating the efficacy and safety of NEPA, a fixed-dose combination of netupitant and palonosetron, for prevention of chemotherapy-induced nausea and vomiting following moderately emetogenic chemotherapy. *Ann Oncol*. 2014;25(7):1328-1333. doi:10.1093/annonc/mdu101.

18. Gao A, Guan S, et al. Prolonged usage of fosaprepitant for prevention of delayed chemotherapy-induced nausea and vomiting (CINV) in patients receiving highly emetogenic chemotherapy. *BMC Cancer*. 2023;23(1):609. doi:10.1186/s12885-023-11070-3.

Appendix 1: ECOG Performance Status Scale (Eastern Cooperative Oncology Group)

Patients should be evaluated for general health status before treatment. An important indicator of general health status is the evaluation of performance status (PS). Performance status is an indicator of a patient's general health status and ability to tolerate treatment based on their physical strength. The Eastern Cooperative Oncology Group (ECOG) developed a simplified performance status score table, as shown in the table below:

ECOG performance status scoring criteria

GRADE	ECOG PERFORMANCE STATUS
0	Fully active, able to carry on all pre-disease performance without restriction
1	Restricted in physically strenuous activity but ambulatory and able to carry out work of a light or sedentary nature, e.g., light house work, office work
2	Ambulatory and capable of all selfcare but unable to carry out any work activities; up and about more than 50% of waking hours
3	Capable of only limited selfcare; confined to bed or chair more than 50% of waking hours
4	Completely disabled; cannot carry on any selfcare; totally confined to bed or chair
5	Dead

Appendix 2: NCCN Emetic Risk Classification for Anticancer Agents

EMETOGENIC POTENTIAL OF PARENTERAL ANTICANCER AGENTS

LEVEL	AGENT		
High emetic risk (>90% frequency of emesis)	• AC combination deined as any chemotherapy regimen that contains an anthracycline and cyclophosphamide<br>• Carboplatin AUC ≥4<br>• Carmustine >250 mg/m2	• Cisplatin<br>• Cyclophosphamide >1500 mg/m2<br>• Dacarbazine<br>• Doxorubicin ≥60 mg/m2<br>• Epirubicin >90 mg/m2<br>• Fam-trastuzumab deruxtecan-nxki	• Ifosfamide ≥2 g/m2 per dose<br>• Mechlorethamine<br>• Melphalan ≥140 mg/m2<br>• Sacituzumab govitecan-hziy<br>• Streptozocin
Moderate emetic risk (>30%–90% frequency of emesis)	• Aldesleukin >12–15 million IU/m2<br>• Amifostine >300 mg/m2<br>• Bendamustine<br>• Busulfan<br>• Carboplatin AUC <4<br>• Carmustine ≤250 mg/m2<br>• Clofarabine<br>• Cyclophosphamide ≤1500 mg/m2<br>• Cytarabine >200 mg/m2<br>• Dactinomycin<br>• Daunorubicin	• Dinutuximab<br>• Doxorubicin <60 mg/m2<br>• Dual-drug liposomal encapsulation of cytarabine and daunorubicin<br>• Epirubicin ≤90 mg/m2<br>• Idarubicin<br>• Ifosfamide <2 g/m2 per dose<br>• Irinotecan<br>• Irinotecan (liposomal)<br>• Lurbinectedin	• Melphalan <140 mg/m2<br>• Methotrexate ≥250 mg/m2<br>• Mirvetuximab soravtansine-gynx<br>• Naxitamab-gqgk<br>• Oxaliplatin<br>• Romidepsin<br>• Temozolomide<br>• Trabectedin

Table framework is based on the emetogenicity classifications described in the following publications: Hesketh PJ, et al. J Clin Oncol 1997;15:103-109. Grunberg SM, et al. Support Care Cancer 2011;19:S43-S47.

Appendix 3: Serious Adverse Event (SAE) Report Form

(Standardized template based on ICH-GCP guidelines)

<table>
<tr><td>Report Type</td><td colspan="2">__Initial __Follow-up __Final</td><td colspan="2">Report Date: Year Month Day</td></tr>
<tr><td>Medical Institution & Specialty Name</td><td colspan="2"></td><td>Telephone</td><td></td></tr>
<tr><td>Reporting Unit Name</td><td colspan="2"></td><td>Telephone</td><td></td></tr>
<tr><td rowspan="2">Investigational Drug Name</td><td colspan="4">Chinese Name:</td></tr>
<tr><td colspan="4">English Name:</td></tr>
<tr><td>Drug Category</td><td colspan="3">__Traditional Chinese Medicine
__Chemical Drug __New Biological Product __Radiopharmaceutical
__Imported Drug __Others</td><td>Class II</td></tr>
<tr><td>Clinical Research Phase</td><td colspan="3">__Phase I __Phase II __Phase III __Phase IV
__Bioequivalence Trial __Clinical Verification</td><td>Dosage Form:</td></tr>
<tr><td rowspan="2">Subject Information</td><td>Name:</td><td>Gender:</td><td>Date of Birth:</td><td>Ethnicity:</td></tr>
<tr><td colspan="4">Disease Diagnosis:</td></tr>
<tr><td>SAE Details</td><td colspan="4">__Hospitalization __Prolonged Hospital Stay __Disability __Functional Impairment
__Congenital Anomaly __Life-threatening or Death __Others</td></tr>
<tr><td>SAE Occurrence Time:</td><td colspan="4">SAE Severity: __Mild __Moderate __Severe</td></tr>
</table>

Actions Taken for Investigational Drug	__Continued Use __Dose Reduction __Drug Suspended then Resumed __Drug Discontinued
SAE Outcome	__Symptoms Disappeared (With/Without Sequelae) __Symptoms Persist __Death (Time of Death:)
Relationship Between SAE and Investigational Drug	__Definitely Related __Possibly Related __Possibly Unrelated __Unrelated __Unassessable
SAE Reporting Status	Domestic: __Yes __No __Unknown International: __Yes __No __Unknown
Detailed Description of SAE Occurrence and Handling:	

Appendix 4: NCI CTCAE 5.0

Gastrointestinal disorders					
**CTCAE Term**	**Grade 1**	**Grade 2**	**Grade 3**	**Grade 4**	**Grade 5**
Diarrhea	Increase of <4 stools per day over baseline; mild increase in ostomy output compared to baseline	Increase of 4 - 6 stools per day over baseline; moderate increase in ostomy output compared to baseline; limiting instrumental ADL	Increase of >=7 stools per day over baseline; hospitalization indicated; severe increase in ostomy output compared to baseline; limiting self care ADL	Life-threatening consequences; urgent intervention indicated	Death
**Definition:** A disorder characterized by an increase in frequency and/or loose or watery bowel movements.<br>**Navigational Note:** -					
Dry mouth	Symptomatic (e.g., dry or thick saliva) without significant dietary alteration; unstimulated saliva flow >0.2 ml/min	Moderate symptoms; oral intake alterations (e.g., copious water, other lubricants, diet limited to purees and/or soft, moist foods); unstimulated saliva 0.1 to 0.2 ml/min	Inability to adequately aliment orally; tube feeding or TPN indicated; unstimulated saliva <0.1 ml/min	-	-

**Definition:** A disorder characterized by reduced salivary flow in the oral cavity.<br>**Navigational Note:** -					
Duodenal fistula	Asymptomatic	Symptomatic, invasive intervention not indicated	Invasive intervention indicated	Life-threatening consequences; urgent intervention indicated	Death
**Definition:** A disorder characterized by an abnormal communication between the duodenum and another organ or anatomic site.<br>**Navigational Note:** -					
Duodenal hemorrhage	Mild symptoms; intervention not indicated	Moderate ymptoms; intervention indicated	Transfusion indicated; invasive intervention indicated; hospitalization	Life-threatening consequences; urgent intervention indicated	Death
**Definition:** A disorder characterized by bleeding from the duodenum.<br>**Navigational Note:** -					
Duodenal obstruction	Asymptomatic; clinical or diagnostic observations only; intervention not indicated	Symptomatic; altered GI function	Hospitalization indicated; invasive intervention indicated	Life-threatening consequences; urgent operative intervention indicated	Death
**Definition:** A disorder characterized by blockage of the normal flow of stomach contents through the duodenum.<br>**Navigational Note:** -					

**Gastrointestinal disorders**					
**CTCAE Term**	**Grade 1**	**Grade 2**	**Grade 3**	**Grade 4**	**Grade 5**
Colonic obstruction	Asymptomatic; clinical or diagnostic observations only; intervention not indicated	Symptomatic; altered GI function	Hospitalization indicated; invasive intervention indicated	Life-threatening consequences; urgent operative intervention indicated	Death
**Definition:** A disorder characterized by blockage of the normal flow of the intestinal contents in the colon.<br>**Navigational Note:** -					
Colonic perforation	-	Invasive intervention not indicated	Invasive intervention indicated	Life-threatening consequences; urgent intervention indicated	Death
**Definition:** A disorder characterized by a rupture in the colonic wall.<br>**Navigational Note:** -					
Colonic stenosis	Asymptomatic; clinical or diagnostic observations only; intervention not indicated	Symptomatic; altered GI function	Severely altered GI function; tube feeding or hospitalization indicated; elective operative intervention indicated	Life-threatening consequences; urgent operative intervention indicated	Death
**Definition:** A disorder characterized by a narrowing of the lumen of the colon.					

**Navigational Note:** -					
Colonic ulcer	Asymptomatic; clinical or diagnostic observations only; intervention not indicated	Symptomatic; altered GI function	Severely altered GI function; TPN indicated; elective invasive intervention indicated	Life-threatening consequences; urgent operative intervention indicated	Death
**Definition:** A disorder characterized by a circumscribed, erosive lesion on the mucosal surface of the colon.					
**Navigational Note:** -					
Constipation	Occasional or intermittent symptoms; occasional use of stool softeners, laxatives, dietary modification, or enema	Persistent symptoms with regular use of laxatives or enemas; limiting instrumental ADL	Obstipation with manual evacuation indicated; limiting self care ADL	Life-threatening consequences; urgent intervention indicated	Death
**Definition:** A disorder characterized by irregular and infrequent or difficult evacuation of the bowels.					
**Navigational Note:** -					
Dental caries	One or more dental caries, not involving the root	Dental caries involving the root	Dental caries resulting in pulpitis or periapical abscess or resulting in tooth loss	-	-
**Definition:** A disorder characterized by the decay of a tooth, in which it becomes softened, discolored and/or porous.					
**Navigational Note:** -					

**Gastrointestinal disorders**					
**CTCAE Term**	**Grade 1**	**Grade 2**	**Grade 3**	**Grade 4**	**Grade 5**
Anal pain	Mild pain	Moderate pain; limiting instrumental ADL	Severe pain; limiting self care ADL	-	-
**Definition:** A disorder characterized by a sensation of marked discomfort in the anal region. **Navigational Note:** -					
Anal stenosis	Asymptomatic; clinical or diagnostic observations only; intervention not indicated	Symptomatic; altered GI function	Symptomatic and severely altered GI function; non-emergent operative intervention indicated; TPN or hospitalization indicated	Life-threatening consequences; urgent operative intervention indicated	Death
**Definition:** A disorder characterized by a narrowing of the lumen of the anal canal. **Navigational Note:** -					
Anal ulcer	Asymptomatic; clinical or diagnostic observations only; intervention not indicated	Symptomatic; altered GI function	Severely altered GI function; TPN indicated; elective invasive intervention	Life-threatening consequences; urgent operative intervention indicated	Death
**Definition:** A disorder characterized by a circumscribed, erosive lesion on the mucosal surface of the anal canal. **Navigational Note:** -					
Ascites	Asymptomatic; clinical or	Symptomatic; medical intervention indicated	Severe symptoms; invasive intervention indicated	Life-threatening	Death

	diagnostic observations only; intervention not indicated			consequences; urgent operative intervention indicated	
**Definition:** A disorder characterized by accumulation of serous or hemorrhagic fluid in the peritoneal cavity.<br>**Navigational Note:** -					
Belching	Increase from baseline	Intervention initiated (including over the counter medications)	-	-	-
**Definition:** To expel gasnoisily from the mouth.<br>**Navigational Note:** Synonym: Burping					
Bloating	No change in bowel function or oral intake	Symptomatic, decreased oral intake; change in bowel function	-	-	-
**Definition:** A disorder characterized by subject-reported feeling of uncomfortable fullness of the abdomen.<br>**Navigational Note:** -					

**Psychiatric disorders**					
**CTCAE Term**	**Grade 1**	**Grade 2**	**Grade 3**	**Grade 4**	**Grade 5**
Agitation	Mild mood alteration	Moderate mood alteration	Severe agitation; hospitalization not indicated	Life-threatening consequences; urgent intervention indicated	-

**Definition:** A disorder characterized by a state of restlessness associated with unpleasant feelings of irritability and tension.<br>**Navigational Note:** -					
Anorgasmia	Inability to achieve orgasm not adversely affecting	Inability to achieve orgasm adversely affecting relationship	-	-	-
**Definition:** A disorder characterized by an inability to achieve orgasm.<br>**Navigational Note:** -					
Anxiety	Mild symptoms; intervention not indicated	Moderate symptoms; limiting instrumental ADL	Severe symptoms; limiting self care ADL; hospitalization indicated	Life-threatening consequences; urgent intervention indicated	-
**Definition:** A disorder characterized by apprehension of danger and dread accompanied by restlessness, tension, tachycardia, and dyspnea unattached to a clearly identifiable stimulus.<br>**Navigational Note:** -					
Confusion	Mild disorientation	Moderate disorientation; limiting instrumental ADL	Severe disorientation; limiting self care ADL	Life-threatening consequences; urgent intervention indicated	-
**Definition:** A disorder characterized by a lack of clear and orderly thought and behavior.<br>**Navigational Note:** -					

Delayed orgasm	Delay in achieving orgasm not adversely affecting	Delay in achieving orgasm adversely affecting relationship	-	-	-
**Definition:** A disorder characterized by sexual dysfunction characterized by a delay in climax.<br>**Navigational Note:** -					
Delirium	Mild acute confusional state	Moderate and acute confusional state; limiting instrumental ADL	Severe and acute confusional state; limiting self care ADL; urgent intervention indicated; new onset	Life-threatening consequences, threats of harm to self or others; urgent intervention indicated	Death
**Definition:** A disorder characterized by the acute and sudden development of confusion, illusions, movement changes, inattentiveness, agitation, and hallucinations. Usually, it is a reversible condition.<br>**Navigational Note:** -					
Delusions	-	Moderate delusional symptoms	Severe delusional symptoms; hospitalization not indicated; new onset	Life-threatening consequences, threats of harm to self or others; hospitalization indicated	Death
**Definition:** A disorder characterized by false personal beliefs held contrary to reality, despite contradictory evidence and common sense.<br>**Navigational Note:** -					

**Psychiatric disorders**

CTCAE Term	Grade 1	Grade 2	Grade 3	Grade 4	Grade 5
Depression	Mild depressive symptoms	Moderate depressive symptoms; limiting instrumental ADL	Severe depressive symptoms; limiting self care ADL; hospitalization not indicated	Life-threatening consequences, threats of harm to self or others; hospitalization indicated	Death
**Definition:** A disorder characterized by melancholic feelings of grief or unhappiness. **Navigational Note:** -					
Euphoria	Mild mood elevation	Moderate mood elevation	Severe mood elevation (e.g., hypomania)	-	-
**Definition:** A disorder characterized by an exaggerated feeling of well-being which is disproportionate to events and stimuli. **Navigational Note:** -					
Hallucinations	Mild hallucinations (e.g., perceptual distortions)	Moderate hallucinations	Severe hallucinations; hospitalization not indicated	Life-threatening consequences, threats of harm to self or others; hospitalization indicated	Death
**Definition:** A disorder characterized by a false sensory perception in the absence of an external stimulus. **Navigational Note:** -					
Insomnia	Mild difficulty falling asleep, staying asleep or waking up early	Moderate difficulty falling asleep, staying asleep or waking up early	Severe difficulty in falling asleep, staying	-	-

			asleep or waking up early		
**Definition:** A disorder characterized by difficulty in falling asleep and/or remaining asleep. **Navigational Note:** -					
Irritability	Mild; easily consolable	Moderate; limiting instrumental ADL; increased attention indicated	Severe abnormal or excessive response; limiting self care ADL; inconsolable; medical or psychiatric intervention indicated	-	-
**Definition:** A disorder characterized by an abnormal responsiveness to stimuli or physiological arousal; may be in response to pain, fright, a drug, an emotional situation or a medical condition. **Navigational Note:** -					
Libido decreased	Decrease in sexual interest not adversely affecting relationship	Decrease in sexual interest adversely affecting relationship	-	-	-
**Definition:** A disorder characterized by a decrease in sexual desire. **Navigational Note:** -					
Libido increased	Present	-	-	-	-
**Definition:** A disorder characterized by an increase in sexual desire. **Navigational Note:** -					

**Psychiatric disorders**					
**CTCAE Term**	**Grade 1**	**Grade 2**	**Grade 3**	**Grade 4**	**Grade 5**
Mania	Mild manic symptoms (e.g., elevated mood, rapid thoughts, rapid speech, decreased need for sleep)	Moderate manic symptoms (e.g., relationship and work difficulties; poor hygiene)	Severe manic symptoms (e.g., hypomania; major sexual or financial indiscretions); hospitalization not indicated; new onset	Life-threatening consequences, threats of harm to self or others; hospitalization indicated	Death
**Definition:** A disorder characterized by excitement of psychotic proportions manifested by mental and physical hyperactivity, disorganization of behavior and elevation of mood.<br>**Navigational Note:** -					
Personality change	Mild personality change	Moderate personality change	Severe personality change; hospitalization not indicated	Life-threatening consequences, threats of harm to self or others; hospitalization indicated	-
**Definition:** A disorder characterized by a conspicuous change in a person's behavior and thinking.<br>**Navigational Note:** -					
Psychosis	Mild psychotic symptoms	Moderate psychotic symptoms (e.g., disorganized speech; impaired reality testing)	Severe psychotic symptoms (e.g., paranoid, extreme disorganization); hospitalization not indicated; new onset	Life-threatening consequences, threats of harm to self or others; hospitalization indicated	Death

**Definition:** A disorder characterized by personality change, impaired functioning, and loss of touch with reality. It may be a manifestation of schizophrenia, bipolar disorder or brain tumor.<br>**Navigational Note:** -					
Restlessness	Mild symptoms; intervention not indicated	Moderate symptoms; limiting instrumental ADL	Severe symptoms; limiting selfcare ADL	-	-
**Definition:** A disorder characterized by an inability to rest, relax or be still.<br>**Navigational Note:** -					
Suicidal ideation	Increased thoughts of death but no wish to kill oneself	Suicidal ideation with no specific plan or intent	Specific plan to commit suicide without serious intent to die which may not require hospitalization	Specific plan to commit suicide with serious intent to die which requires hospitalization	-
**Definition:** A disorder characterized by thoughts of taking one's own life.<br>**Navigational Note:** -					
Suicide attempt	-	-	Suicide attempt or gesture without intent to die	Suicide attempt with intent to die which requires hospitalization	Death
**Definition:** A disorder characterized by self-inflicted harm in an attempt to end one's own life.<br>**Navigational Note:** -					

**Immune system disorders**

CTCAE Term	Grade 1	Grade 2	Grade 3	Grade 4	Grade 5
Allergic reaction	Systemic intervention not indicated	Oral intervention indicated	Bronchospasm; hospitalization indicated for clinical sequelae; intravenous intervention indicated	Life-threatening consequences; urgent intervention indicated	Death
**Definition:** A disorder characterized by an adverse local or general response from exposure to an allergen.<br>**Navigational Note:** If related to infusion, use Injury, poisoning and procedural complications: Infusion related reaction. Do not report both.					
Anaphylaxis	-	-	Symptomatic bronchospasm, with or without urticaria; parenteral intervention indicated; allergy-related edema/angioedema; hypotension	Life-threatening consequences; urgent intervention indicated	Death
**Definition:** A disorder characterized by an acute inflammatory reaction resulting from the release of histamine and histamine-like substances from mast cells, causing a hypersensitivity immune response. Clinically, it presents with breathing difficulty, dizziness, hypotension, cyanosis and loss of consciousness and may lead to death.<br>**Navigational Note:** -					
Autoimmune disorder	Asymptomatic; serologic or other evidence of autoimmune reaction, with normal organ	Evidence of autoimmune reaction involving a non-essential organ or function (e.g., hypothyroidism)	Autoimmune reactions involving major organ (e.g., colitis, anemia, myocarditis, kidney)	Life-threatening consequences; urgent intervention indicated	Death

	function; intervention not indicated				
**Definition:** A disorder characterized by loss of function or tissue destruction of an organ or multiple organs, arising from humoral or cellular immune responses of the individual to his own tissue constituents.<br>**Navigational Note:** Prior to using this term consider specific autoimmune AEs					
Cytokine release syndrome	Fever with or without constitutional symptoms	Hypotension responding to fluids; hypoxia responding to <40% O2	Hypotension managed with one pressor; hypoxia requiring ≥ 40% O2	Life-threatening consequences; urgent intervention indicated	Death
**Definition:** A disorder characterized by fever, tachypnea, headache, tachycardia, hypotension, rash, and/or hypoxia caused by the release of cytokines.<br>**Navigational Note:** Also consider reporting other organ dysfunctions including neurological toxicities such as: Psychiatric disorders: Hallucinations or Confusion; Nervous system disorders: Seizure, Dysphasia, Tremor, or Headache					
Serum sickness	Asymptomatic; clinical or diagnostic observations only;	Moderate arthralgia; fever, rash, urticaria, antihistamines indicated	Severe arthralgia or arthritis; extensive rash; steroids or IV fluids indicated	Life-threatening consequences; pressor or ventilatory support indicated	Death
**Definition:** A disorder characterized by a delayed-type hypersensitivity reaction to foreign proteins derived from an animal serum. It occurs approximately six to twenty-one days following the administration of the foreign antigen. Symptoms include fever, arthralgias, myalgias, skin eruptions, lymphadenopathy, chest marked discomfort and dyspnea.<br>**Navigational Note:** -					

**Nervous system disorders**					
**CTCAE Term**	**Grade 1**	**Grade 2**	**Grade 3**	**Grade 4**	**Grade 5**
Encephalopathy	Mild symptoms	Moderate symptoms; limiting instrumental ADL	Severe symptoms; limiting self care ADL	Life-threatening consequences; urgent intervention indicated	Death
**Definition:** A disorder characterized by a pathologic process involving the brain.<br>**Navigational Note:** -					
Extrapyramidal disorder	Mild involuntary movements	Moderate involuntary movements; limiting instrumental ADL	Severe involuntary movements or torticollis; limiting self care ADL	Life-threatening consequences; urgent intervention indicated	Death
**Definition:** A disorder characterized by abnormal, repetitive, involuntary muscle movements, frenzied speech and extreme restlessness.<br>**Navigational Note:** Synonym: Restless legs					
Facial muscle weakness	Asymptomatic; clinical or diagnostic observations only; intervention not indicated	Moderate symptoms; limiting instrumental ADL	Severe symptoms; limiting self care ADL	-	-
**Definition:** A disorder characterized by a reduction in the strength of the facial muscles.<br>**Navigational Note:** -					

Facial nerve disorder	Asymptomatic; clinical or diagnostic observations only; intervention not indicated	Moderate symptoms; limiting instrumental ADL	Severe symptoms; limiting self care ADL	-	-
**Definition:** A disorder characterized by dysfunction of the facial nerve (seventh cranial nerve).<br>**Navigational Note:** -					
Glossopharyngeal nerve disorder	Asymptomatic; clinical or diagnostic observations only; intervention not indicated	Moderate symptoms; limiting instrumental ADL	Severe symptoms; limiting self care ADL	Life-threatening consequences; urgent intervention indicated	Death
**Definition:** A disorder characterized by dysfunction of the glossopharyngeal nerve (ninth cranial nerve). **Navigational Note:** -					
Guillain-Barre syndrome	Mild symptoms	Moderate symptoms; limiting instrumental ADL	Severe symptoms; limiting self care ADL	Life-threatening consequences; urgent intervention indicated; intubation	Death
**Definition:** A disorder characterized by the body's immune system attacking the peripheral nervous system causing ascending paralysis.<br>**Navigational Note:** -					
Headache	Mild pain	Moderate pain; limiting instrumental ADL	Severe pain; limiting self care	-	-
**Definition:** A disorder characterized by a sensation of marked discomfort in various parts of the head, not confined to the area of distribution of any nerve.<br>**Navigational Note:** -					

**Metabolism and nutrition disorders**					
**CTCAE Term**	**Grade 1**	**Grade 2**	**Grade 3**	**Grade 4**	**Grade 5**
Glucose intolerance	Asymptomatic; clinical or diagnostic observations only; intervention not indicated	Symptomatic; dietary modification or oral agent indicated	Severe symptoms; insulin indicated	Life-threatening consequences; urgent intervention indicated	Death
**Definition:** A disorder characterized by an inability to properly metabolize glucose.<br>**Navigational Note:** -					
Hypercalcemia	Corrected serum calcium of >ULN - 11.5 mg/dL; >ULN - 2.9 mmol/L; Ionized calcium >ULN - 1.5 mmol/L	Corrected serum calcium of >11.5 - 12.5 mg/dL; >2.9 - 3.1 mmol/L; Ionized calcium >1.5 - 1.6 mmol/L; symptomatic	Corrected serum calcium of >12.5 - 13.5 mg/dL; >3.1 - 3.4 mmol/L; Ionized calcium >1.6 - 1.8 mmol/L; hospitalization indicated	Corrected serum calcium of >13.5 mg/dL; >3.4 mmol/L; Ionized calcium >1.8 mmol/L; life-threatening consequences	Death
**Definition:** A disorder characterized by laboratory test results that indicate an elevation in the concentration of calcium (corrected for albumin) in blood.<br>**Navigational Note:** -					
