# Supplementary material for: Phase III Trial: Single Low-Dose 5 mg Dexamethasone with NEPA for Preventing 168 h Nausea and Vomiting of Diverse Highly or Moderately Emetogenic Chemotherapy (LD-NEPA)
Source: Diseases. 2026 Jun 27;14(7):231. doi: 10.3390/diseases14070231 (PMC13407607; doi:10.3390/diseases14070231)
Supplement: Supplementary file 1 [file diseases-14-00231-s001.zip › Supplementary Table.pdf]

Table S1. Treatment-Related Adverse Events.

Treatment-Related Adverse Events [b]	No. (%) of participants								
	5D Group (*n* = 89)				8D Group (*n* = 91)				
	Grade 1	Grade 2	Grade 3	All Grades	Grade 1	Grade 2	Grade 3	All Grades	*p-value* [a]
Indigestion /Heartburn or Reflux	24 (27.0)	0	0	24 (27.0)	24 (26.4)	0	0	24 (26.4)	0.93
Insomnia	6 (6.7)	0	0	6 (6.7)	7 (7.7)	0	0	7 (7.7)	0.81
Hiccups	18 (20.2)	0	0	18 (20.2)	23 (25.3)	0	0	23 (25.3)	0.42
Agitation	5 (5.6)	0	0	5 (5.6)	11 (12.1)	0	0	11 (12.1)	0.13
Facial rash/Acne	4 (4.5)	2 (2.2)	0	6 (6.7)	5 (5.5)	3 (3.3)	0	8 (8.8)	0.61
Headache	8 (9.0)	0	0	8 (9.0)	3 (3.3)	0	0	3 (3.3)	0.11
Constipation	16 (18.0)	3 (3.4)	0	19 (21.4)	24 (26.4)	2 (2.2)	0	26 (28.6)	0.30
Diarrhea	14 (15.7)	0	1 (1.1)	15 (16.8)	13 (14.3)	7 (7.7)	0	20 (22.0)	0.31
Hyperglycemia [c]	25 (25.2)	1 (1.5)	0	26 (26.7)	24 (32.9)	2 (2.7)	0	26 (35.6)	0.79
QTcB exceeds 500 ms [d]				1 (1.4)				2 (3.1)	0.61
QTcB increase exceeds 60 ms[d]				2 (2.9)				4 (6.2)	0.43

Abbreviations: 5D, 5 mg dexamethasone plus NEPA; 8D, 8 mg dexamethasone plus NEPA; QTcB, QT interval corrected using Bazett's formula.

a: *p* value indicates outcomes from the Mann-Whitney U test. Statistical significance was defined as $p < 0.05$ (two-sided).

b: All treatment-related adverse events (TRAEs) were analyzed in the modified intention-to-treat (mITT) population, except for hyperglycemia and cardiotoxicity, which were assessed in the per-protocol set (PPS).

c: Complete glucose data were available for 150 patients (5D=77, 8D=73).

d: ECG abnormalities (all-grade) were documented in 134 patients (5D=69, 8D=65).

Table S2. Steroid-associated adverse events during treatment.

Adverse event (Grade 1–3) [b]	No. (%) of participants		p-value[a]
	5D Group (n = 89)	8D Group (n = 91)	
Hyperglycemia [c]	26 (26.7)	26 (35.6)	0.81
Facial rash/Acne	6 (6.7)	8 (8.8)	0.61
Insomnia	6 (6.7)	7 (7.7)	0.81
Hiccups	18 (20.2)	23 (25.3)	0.42
Agitation	5 (5.6)	11 (12.1)	0.13
Headache	8 (9.0)	3 (3.3)	0.11
Constipation	19 (21.4)	26 (28.6)	0.26
Diarrhea	15 (16.8)	20 (22.0)	0.39

Abbreviations: 5D, 5 mg dexamethasone plus NEPA; 8D, 8 mg dexamethasone plus NEPA.

a: p value indicates outcomes from the Chi-square test. Statistical significance was defined as $p < 0.05$ (two-sided).

b: All treatment-related adverse events (TRAEs) were analyzed in the modified intention-to-treat (mITT) population, except for hyperglycemia, which were assessed in the per-protocol set (PPS).

c: Complete glucose data were available for 150 patients (5D=77, 8D=73).

Table S3. Complete response rates stratified by emetogenic risk category (HEC vs MEC)**.**

Risk	5D Group (n = 89)	8D Group (n = 91)	RR (95% CI)	p-value *
MEC	61/78 (78.2%)	51/73 (69.9%)	1.12 (0.93-1.36)	0.268
HEC	9/11 (81.8%)	13/18 (72.2%)	1.13 (0.76-1.69)	0.677

Abbreviations: MEC, moderately emetogenic chemotherapy; HEC, highly emetogenic chemotherapy; 5D, 5 mg dexamethasone plus NEPA; 8D, 8 mg dexamethasone plus NEPA; RR, risk ratios; CI, confidence intervals.

*: $p < 0.05$ was considered statistically significant.
