# Supplementary figures and images for: Phase III Trial: Single Low-Dose 5 mg Dexamethasone with NEPA for Preventing 168 h Nausea and Vomiting of Diverse Highly or Moderately Emetogenic Chemotherapy (LD-NEPA)
Source: Diseases. 2026 Jun 27;14(7):231. doi: 10.3390/diseases14070231 (PMC13407607; doi:10.3390/diseases14070231)

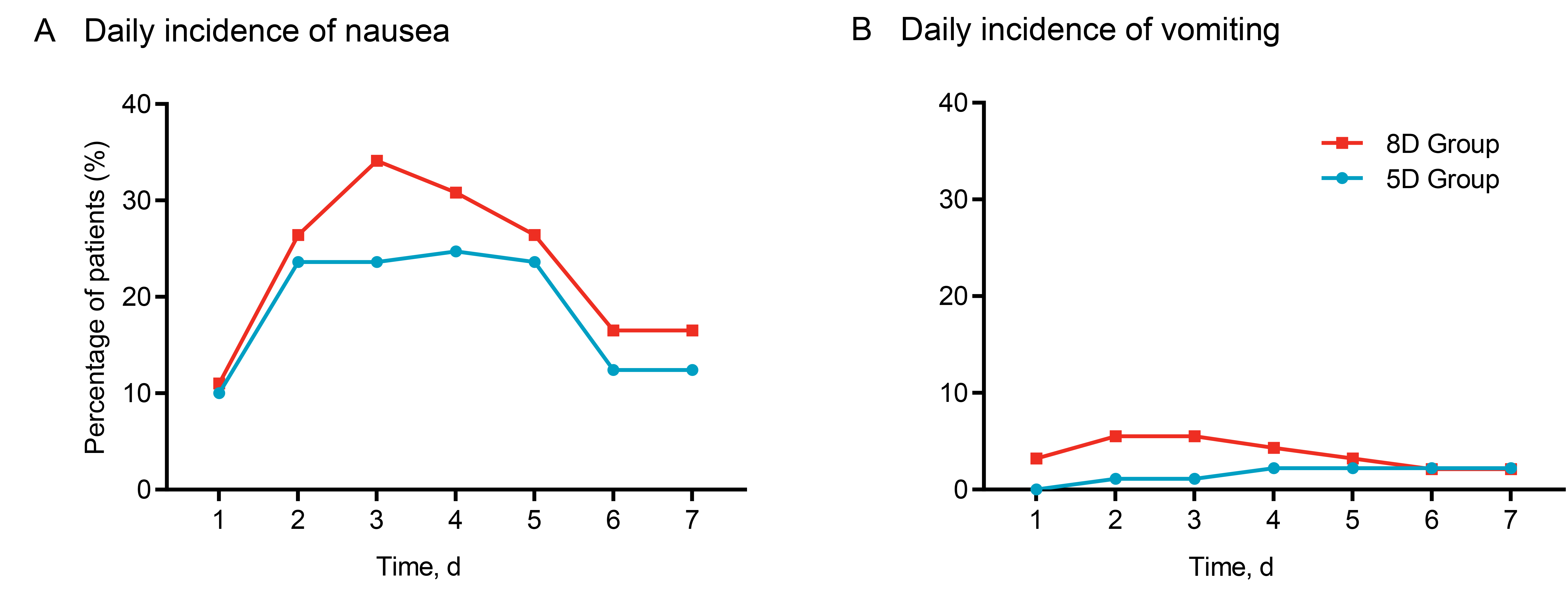

Supplement: Supplementary file 1 [file diseases-14-00231-s001.zip › Supplementary Figure S1.tif]

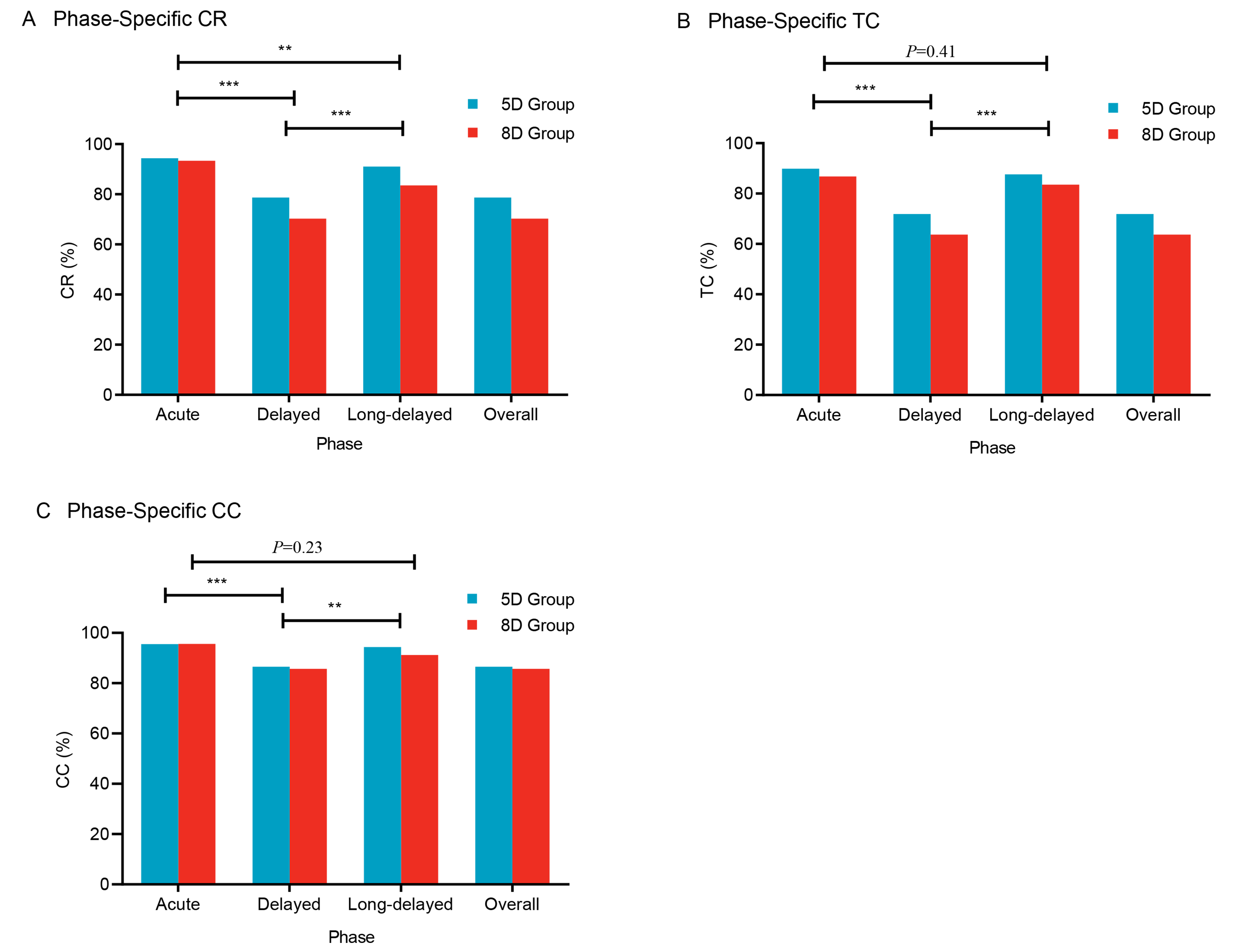

Supplement: Supplementary file 1 [file diseases-14-00231-s001.zip › Supplementary Figure S2.tif]
